# From Cell Lines to Patients: Dissecting the Proteomic Landscape of Exosomes in Breast Cancer

**DOI:** 10.3390/diagnostics15081028

**Published:** 2025-04-17

**Authors:** Aleksei Shefer, Lyudmila Yanshole, Ksenia Proskura, Oleg Tutanov, Natalia Yunusova, Alina Grigor’eva, Yuri Tsentalovich, Svetlana Tamkovich

**Affiliations:** 1 Laboratory of Molecular Medicine, Institute of Chemical Biology and Fundamental Medicine, Siberian Branch of the Russian Academy of Sciences, 630090 Novosibirsk, Russia; a.shefer@g.nsu.ru (A.S.);; 2 Institute of Medicine and Medical Technologies, Novosibirsk State University, 630090 Novosibirsk, Russia; 3 Laboratory of Proteomics and Metabolomics, International Tomography Center, Siberian Branch of Russian Academy of Sciences, 630090 Novosibirsk, Russia; lucy@tomo.nsc.ru (L.Y.); yura@tomo.nsc.ru (Y.T.); 4 Department of Mammology, Novosibirsk Regional Clinical Oncological Dispensary, 630108 Novosibirsk, Russia; 5 Department of Medicine, Vanderbilt University Medical Center, Nashville, TN 37203, USA; ostutanov@gmail.com; 6 Laboratory of Tumor Biochemistry, Cancer Research Institute, Tomsk National Research Medical Center, Russian Academy of Science, 634028 Tomsk, Russia; bochkarevanv@oncology.tomsk.ru; 7 Institute of Oncology and Neurosurgery, E. Meshalkin National Medical Research Center of the Ministry of Health of the Russian Federation, 630090 Novosibirsk, Russia

**Keywords:** exosomes, extracellular vesicles, MALDI-TOF mass spectrometry, bioinformatics analysis, HUVEC, MCF-7, BT-474, breast cancer patients, plasma, blood

## Abstract

**Background:** Breast cancer (BC) is the most common cancer among women worldwide; therefore, the efforts of many scientists are aimed at finding effective biomarkers for this disease. It is known that exosomes are nanosized extracellular vesicles (EVs) that are released from various cell types, including cancer cells. Exosomes are directly involved in governing the physiological and pathological processes of an organism through the horizontal transfer of functional molecules (proteins, microRNA, etc.) from producing to receiving cells. Since the diagnosis and treatment of BC have been improved substantially with exosomes, in this study, we isolated breast carcinoma cell-derived exosomes, primary endotheliocyte-derived exosomes, and blood exosomes from BC patients (BCPs) in the first stage of disease and investigated their proteomic profiles. **Methods**: Exosomes were isolated from the samples by ultrafiltration and ultracentrifugation, followed by mass spectrometric and bioinformatics analyses of the data. The exosomal nature of vesicles was verified using transmission electron microscopy and flow cytometry. **Results**: Exosome proteins secreted by MCF-7 and BT-474 cells were found to form two clusters, one of which enhanced the malignant potential of cancer cells, while the other coincided with a cluster of HUVEC-derived exosome proteins. Despite the different ensembles of proteins in exosomes from the MCF-7 and BT-474 lines, the relevant portions of these proteins are involved in similar biological pathways. Comparison analysis revealed that more BC-associated proteins were found in the exosomal fraction of blood from BCPs than in the exosomal fraction of conditioned medium from cells mimicking the corresponding cancer subtype (89% and 81% for luminal A BC and MCF-7 cells and 86% and 80% for triple-positive BC and BT-474 cells, respectively). **Conclusions:** Tumor-associated proteins should be sought not in exosomes secreted by cell lines but in the composition of blood exosomes from cancer patients, while the contribution of endotheliocyte exosomes to the total pool of blood exosomes can be neglected.

## 1. Introduction

In recent decades, there has been a steady increase in the number of new cases of malignant neoplasms, which is primarily due to increased life expectancy, as well as changes in lifestyle and exposure to unfavorable environmental factors. Today, cancer ranks second in mortality, conceding only to cardiovascular diseases. In 2022, breast cancer (BC) accounted for 23.8% of all new cancer cases in women worldwide. In particular, there were 2.3 million new cases of BC and 666,000 deaths from BC worldwide in 2022 [1]. The incidence of BC continues to increase despite the success of mammographic screening (depending on the country, from 14.2% (Republic of Moldova) to 47.3% (Czech Republic) of patients are diagnosed at stage I) [2]. Obviously, early BC diagnosis will increase the effectiveness of anti-tumor therapy and the survival rate of tumor patients. Thus, there is an urgent need to identify more effective and non-invasive surrogate markers that can guide not only early diagnosis but also the selection of therapeutic strategies for individual patients and the accurate assessment of prognosis [3]. Furthermore, BC is characterized by considerable tissue heterogeneity, showing distinct clinical and biological features, which makes tumors respond differently to treatments and complicates their management [4]. Today, molecular profiles have been largely explored, providing a well-established classification of BCs into five well-settled subtypes: luminal A, luminal B Her2+, luminal B Her2-, basal-like, and human epidermal growth factor receptor 2 (Her2)-enriched [2,5]. These molecular subtypes of BC are established by tumor biopsy, which can cause the displacement of tumor cells, promoting metastasis and various pathological changes in breast tissue [6]. In addition, biopsy exhibits inaccuracy in determining the BC subtype and is not posed to track patient follow-up [4]. In this regard, a promising direction in molecular oncology has been the search for new tumor markers in the composition of extracellular vesicles (EVs) by liquid biopsy. Among EVs, exosomes, small vesicles 30–150 nm in diameter with a lipid bilayer membrane and tetraspanins CD9, CD63, and CD81 on their crown, are prominent [7,8]. The advantage of exosomes is the sorting of biologically active molecules (proteins, different types of RNA) at the maturation stage of these vesicles [9]. Recently, exosomes from breast cancer cell lines have been shown to be a rich source of breast cancer-related proteins and surface biomarkers and can be used for the diagnosis and prognosis of the disease [10,11]. However, when analyzing the content of exosomes circulating in the blood of cancer patients, it is necessary to take into account that in addition to tumor exosomes, exosomes from endotheliocytes and other non-tumor cells, as well as cells from the tumor microenvironment, are present in the blood.

To assess the diagnostic potential of blood exosomes and evaluate the contribution of endotheliocytes to the blood exosome proteome, we performed a differential analysis of exosomal proteomes from primary endotheliocytes, from two cell lines mimicking luminal A and luminal Her2-positive BC, and from the blood of patients with these BC subtypes.

## 2. Materials and Methods

### 2.1. Isolation and Cultivation of Human Umbilical Vein Endothelial Cells (HUVECs)

HUVECs were obtained from three donors. Each vein was washed sequentially with 50 mL phosphate-buffered saline (PBS) and 20 mL collagenase IV buffer (0.1% collagenase IV in buffer containing 1.5 mM HEPES, 14 mM NaCl, 0.4 mM KCl, 0.12 mM CaCl_2_, 0.04 mM MgSO_4_, and 0.76 mM D-glucose, pH 7.4) [12]. The vein was incubated with 0.1% collagenase IV solution at 37 °C for 15 min to release endothelial cells. The collagenase solution containing detached cells was collected and combined with an additional PBS wash of the vein. The pooled solution was centrifuged at 800× *g* for 10 min, and the cell pellet was resuspended in IMDM (Gibco, Aucland, New Zealand) supplemented with 10% fetal bovine serum (FBS) (Thermo Fisher Scientific, Waltham, MA, USA) and penicillin–streptomycin (100 μg/mL) (Thermo Fisher Scientific, Waltham, MA, USA). HUVECs were cultured in a CO_2_ incubator (5% CO_2_) at 37 °C, and adherent cells were washed the next day with fresh IMDM to remove residual blood cells. HUVECs were cultured to 70–80% confluence, and cells from the first passage were used for exosome isolation. For dissociation, cells were treated with 0.1% collagenase IV.

### 2.2. Cancer Cell Line Cultivation

MCF-7 (ATCC^®^ HTB-22™) and BT-474 (ATCC^®^ HTB-20™) BC cell lines were cultured in Dulbecco’s Modified Eagle Medium (DMEM) (Thermo Fisher Scientific, Waltham, MA, USA) supplemented with 10% FBS (Gibco, USA) and penicillin–streptomycin (100 μg/mL) (Thermo Fisher Scientific, Waltham, MA, USA) in a CO_2_ incubator (5% CO_2_) at 37 °C up to 70–80% confluence. Cells were subcultured with a solution of 0.25% trypsin in PBS with 5 mM EDTA.

### 2.3. Isolation of Exosomes from Conditioned Medium

All cells were negative for mycoplasma infection, as confirmed by PCR analysis of the 16S mycoplasma ribosomal gene [13].

FBS was centrifuged at 100,000× *g* for 2 h at 4 °C to remove bovine exosomes. The supernatant was collected and used to prepare bovine exosome-depleted medium. Three days prior to cell harvesting, the culture medium was replaced with a depleted medium containing IMDM for HUVECs or DMEM for MCF-7 and BT-474 cells, a mixture of antibiotics, and 10% FBS devoid of bovine exosomes.

For the isolation of exosomes, after 72 h, conditioned medium was collected and subjected to two successive centrifugations at 300× *g* and 15,000× *g* for 10 min and 20 min, respectively to remove dead cells and cellular debris. To eliminate large EVs, the supernatant was filtered through 100 nm pore-size filters (Minisart high flow, 16553-K, Sartorius, Goettingen, Germany). Exosomes were isolated from the pre-cleared conditioned medium by centrifugation at 100,000× *g* for 90 min at 100,000× *g* at 4 °C. The pellets were suspended in 12 mL of PBS and again centrifuged for 90 min at 100,000× *g* at 4 °C. The washing stage was repeated two times. Then, the supernatant was removed, and exosomes were resuspended in 300 μL of PBS, aliquoted, frozen in liquid nitrogen, and stored at −80 °C.

### 2.4. Ethics Statement

The study protocol was approved by the Ethics Committee of the Institute of Chemical Biology and Fundamental Medicine. Written informed consent was obtained from every female. Human samples were obtained according to the principles expressed in the Declaration of Helsinki. Blood samples from untreated BC patients (BCPs) with from luminal A (n = 5, age range 56–61 years, median age 61) and triple-positive (n = 8, age range 44–69 years, median age 61) subtypes [2,5] (Table 1) were obtained from the Novosibirsk Regional Oncology Dispensary.

### 2.5. Exosome Isolation from Blood

Venous blood (9 mL) was collected by venipuncture in K_3_EDTA spray-coated vacutainers (Improvacuter, Guangzhou, China) and processed within one hour. To isolate total blood exosomes by ultrafiltration and differential ultracentrifugation, a previously described protocol was used [14]. The pellet containing blood exosomes was resuspended in 300 μL of PBS.

### 2.6. Characterization of Exosomes

The morphology and membrane integrity of the isolated exosomes was assessed by transmission electron microscopy (TEM), as described previously [15]. The initial volume of exosomes analyzed using TEM was 15 μL.

To evaluate the protein concentration of exosomes, a NanoOrange Protein Quantitation kit (NanoOrange Protein Quantitation Kit, Molecular Probes, San Jose, CA, USA) was used as described previously [16].

The presence of CD9, CD63, and CD81 tetraspanins in the exosomal crown was confirmed by flow cytometry, as described previously [16]. The median fluorescence intensity (MFI) of stained exosomes was analyzed and compared to the isotype control (BD bioscience, Heidelberg, Germany). Flow cytometry was performed using a Cytoflex instrument (Becman Coulter, BioBay, Suzhou, China) with CytExpert 2.0 Software.

### 2.7. Mass Spectrometry Analysis

For the identification of exosomal proteins by MALDI-TOF mass spectrometry, proteins were separated using SDS-disc electrophoresis, and then fragments of polyacrylamide gel containing the studied proteins were washed from SDS and subjected to trypsinolysis, as described previously [14]. Specifically, samples were applied to the gel in 5 replicates, with line widths of 6 mm each. After the separation of the proteins in the gel, each line was cut into pieces 5 mm thick, resulting in 20 pieces from each line. Peptides from each piece of gel were analyzed independently; a protein was considered reliably identified when it was detected in at least three out of five cases.

Peptide fragments of proteins were extracted from the gel, concentrated, and desalted on C18 ZipTips microcolumns (Milipore, Burlington, MA, USA). Next, 5 μL of a saturated solution of α-cyano-4-hydroxycinnamic acid in 70% acetonitrile was added and then spotted onto an MTP 384 ground steel target plate. After crystallization, the target was loaded into the mass spectrometer to obtain the protein molecular weight spectrum. The acquisition and registration of mass spectra were carried out on an Ultraflex III time-of-flight tandem mass spectrometer (MALDI-TOF/TOF spectrometer) (Bruker Daltonics, Bremen, Germany). To ensure the reliability and reproducibility of the results, all analyses were performed with five biological replicates for each sample.

Spectra were acquired using the following parameters: shots—150, laser frequency—66.7 Hz, laser attenuator offset—85%, laser attenuator range—21%, laser attenuator set—5_ularge, laser focus offset—0%, laser focus range—100%, and laser focus value—4%. The instrument was pre-calibrated for a mass range of 500–3800 kDa. The obtained spectra were converted into mass values using flexAnalysis software 3.4. Protein identification was performed by searching for appropriate candidates in the annotated NCBI and SwissProt databases using the Mascot program (Matrix Science Ltd., London, UK, Available online: www.matrixscience.com/search_form_select.html (accessed on 20 October 2024)) with the following search parameters: species—Homo sapiens, error tolerance—±300 ppm, maximum number of missed cleavages—2, fixed modifications—propionamide (C), variable modifications—oxidation (M), phospho (ST); the identification reliability was not lower than 95%.

The matching of at least two peptides comprising 9 or more amino acid residues was considered a reliable identification of minor proteins [17].

### 2.8. Bioinformatics Analysis

Data from the SwissProt database were translated into the UniProt database for further analysis using the Retrieve/ID mapping platform (Available online: https://www.uniprot.org (accessed on 11 November 2024)). Functional enrichment analysis of exosomal proteomes according to Gene Ontologies was conducted using STRING software 12.0 (Available online: https://www.string-db.org/ (accessed on 10 January 2025)). Cellular localization, molecular functions, and involvement in biological processes were determined using FunRich 3.13 software based on the Gene Ontologies (GO) component, GO function, and GO process categories. Involvement in biological pathways was assessed using the Reactome service (Available online: https://reactome.org/ (accessed on 11 January 2025)). For primary data processing and graph generation, Python 3.11 libraries such as Pandas 2.2.3, Numpy 2.2.0, Matplotlib 3.10.1, and Seaborn 0.13.2 were utilized.

## 3. Results

### 3.1. Characterization of Isolated Exosomes

**Figure 1:**
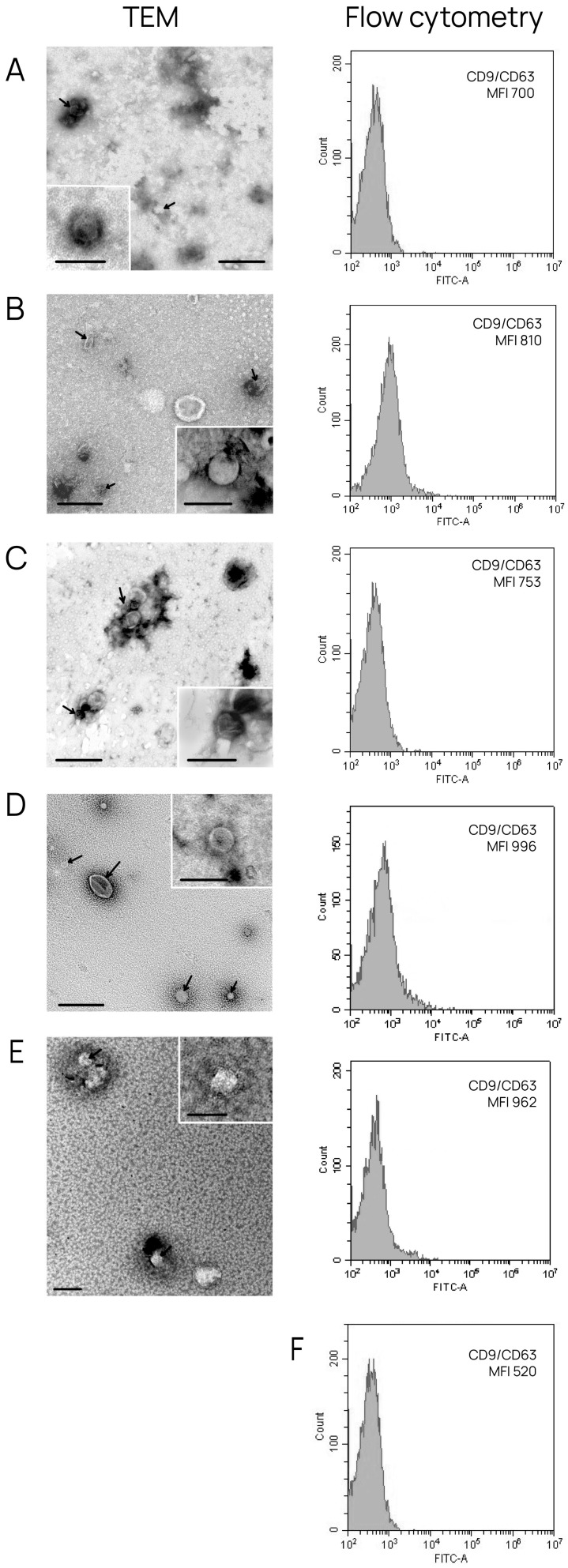
Exosome characterization by TEM and flow cytometry analysis. Overview of exosome preparation and expression of CD63 on CD9-positive vesicles obtained from (**A**) conditioned medium from HUVECs; (**B**) conditioned medium from MCF-7 cells; (**C**) conditioned medium from BT-474 cells; (**D**) blood from patients with luminal A BC; and (**E**) blood from patients with triple-positive BC. Scale bars correspond to 100 nm. Electron microscopy, negative staining by phosphotungstate acid. For flow cytometry, mean MFI values are shown. Negative control (**F**) (latex beads labeled with anti-CD9 and anti-CD63 FITC antibody).

To characterize exosomes isolated from conditioned medium and BCP blood, TEM and flow cytometry were used. TEM revealed the presence of clearly structured cup-shaped vesicles (40–95 nm) of low electron density with preserved membranes in all samples; the portion of vesicles with a size smaller than 30 nm was no more than 13% (Figure 1). We then confirmed that the exosomes isolated from all sources contained typical exosome protein markers, namely, tetraspanins CD9 and CD63. For this purpose, exosomes adsorbed onto aldehyde/sulfate latex beads using anti-CD9 antibodies were stained with FITC-labeled antibodies for the tetraspanin CD63. It was shown that the CD9 and CD63 tetraspanins were comparably represented in all vesicles (Figure 1).

Collectively, the obtained data reveal that the sEVs isolated from conditioned medium and blood have all properties of exosomes [18].

### 3.2. Annotation of Identified Proteins from HUVEC-Derived Exosomes

**Figure 2:**
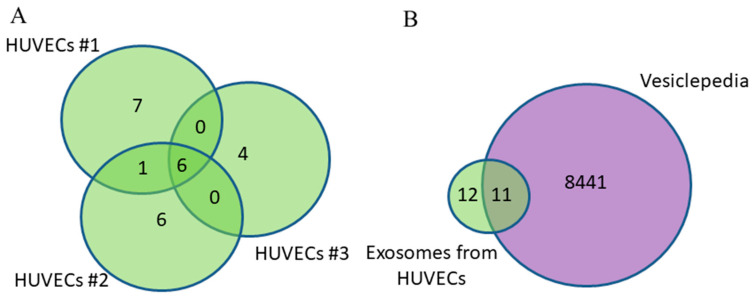
Venn–Euler diagrams of proteins in exosomes from HUVECs from three donors (**A**) and overlap with the Vesiclepedia database (**B**).

Since in addition to tumor exosomes, exosomes from normal cells, including those of endothelial origin, circulate in the blood of cancer patients, the first stage of this work involved the identification and characterization of exosome proteins secreted by primary endothelial cells from three donors. A total of 24 proteins were identified with high confidence (*p* < 0.05) using MALDI-TOF mass spectrometry in the HUVEC-derived exosomes (Table S1); of these, 29% (7 proteins) were common to the three groups (Figure 2A).

The majority (70%) of identified exosomal proteins from HUVECs were previously discovered in other studies using mass spectrometry and were annotated in the Vesiclepedia database [19] (the putative CNGA1-overlapping antisense gene protein is not included in the analysis because its gene is not known). Thus, about one-third of the exosomal proteins are identified in our study for the first time as a part of EVs; previously, they were not annotated in this database (Figure 2B, Table 2).

**Figure 3:**
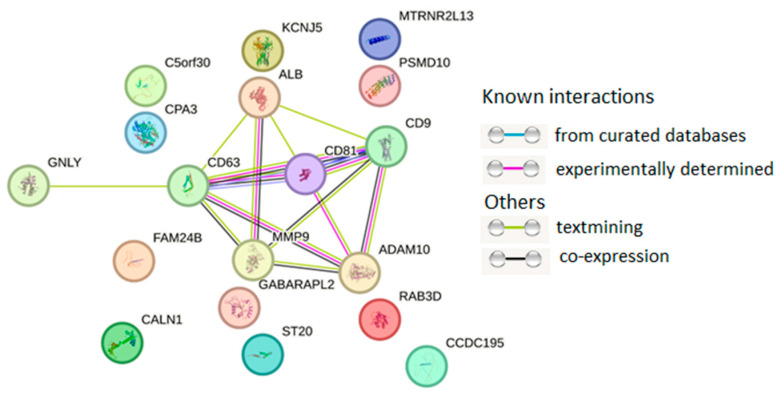
PPI network of 18 proteins from HUVEC-derived exosomes. PPI networks were plotted with STRING (Available online: http://string-db.org/ (accessed on 10 January 2025)) with the following settings: minimum interaction score—high confidence (0.400); active interaction sources—text mining, experiments, databases, co-expression.

The protein–protein interaction (PPI) network was mainly concentrated on the relevance among ALB, CD9, CD63, CD81, ADAM10, and MMP9, all of which are major exosome proteins (Figure 3).

The molecular function GO analysis revealed that HUVEC-derived exosomal proteins were commonly enriched in functions such as “metallopeptidase activity” and “transporter activity” (Table 3).

Six predominant proteins (ALB, CD9, CD63, CD81, MMP9, PSMD10) were found to be significantly (*p* < 0.05) involved in biological pathways (Table 4). In particular, the common proteomic profiles of endothelial exosomes were enriched in terms related to biological processes such as “metabolism of RNA”, “metabolism of mRNA”, “adaptive immune system”, etc.

### 3.3. Annotation of Protein Cargo from BC-Derived Exosomes

**Figure 4:**
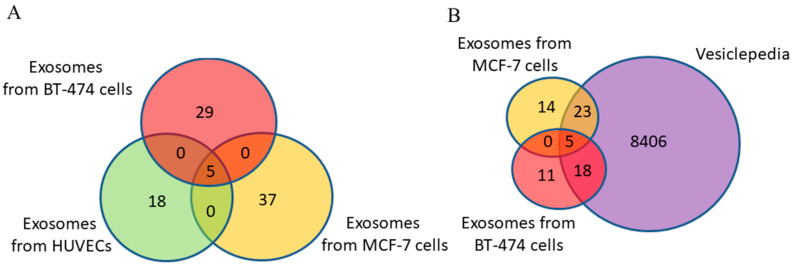
Venn–Euler diagrams of proteins in exosomes from HUVECs, BT-474 cells, and MCF-7 cells (**A**) and overlap with the Vesiclepedia database (**B**).

Using MALDI-TOF mass spectrometry, 34 and 42 proteins were identified with high reliability (*p* < 0.05) in the exosomes from BC cell lines BT-474 and MCF-7, respectively (Supplementary Tables S2 and S3). Of these, only 6% (five proteins) were common to both groups (Supplementary Tables S2 and S3, Figure 4A). It should be noted that the same proteins (CD9, CD24, CD63, CD81, MMP9) were common to exosomes of cancer cells and primary cells.

It should be noted that due to the small size of exosomes (30–150 nm) and their high RNA content, the cargo of these vesicles contains predominantly low-molecular-weight proteins rich in lysine and arginine. As a consequence, the proportion of reliably identified proteins meeting the new criteria for proteome investigation [17] is extremely low (trypsinolysis results in a large number of peptides with fewer than nine amino acid residues).

Approximately 65% of identified exosomal proteins from cancer cells were previously discovered in other studies using mass spectrometry and were annotated in the Vesiclepedia database (Figure 4B) (the putative uncharacterized protein FLJ45840 protein is not included in the analysis because its gene is not known). The non-deposited Vesiclepedia database proteins of exosomes secreted by MCF-7 and BT-474 cells are presented in Table 5 and Table 6, respectively.

**Figure 5:**
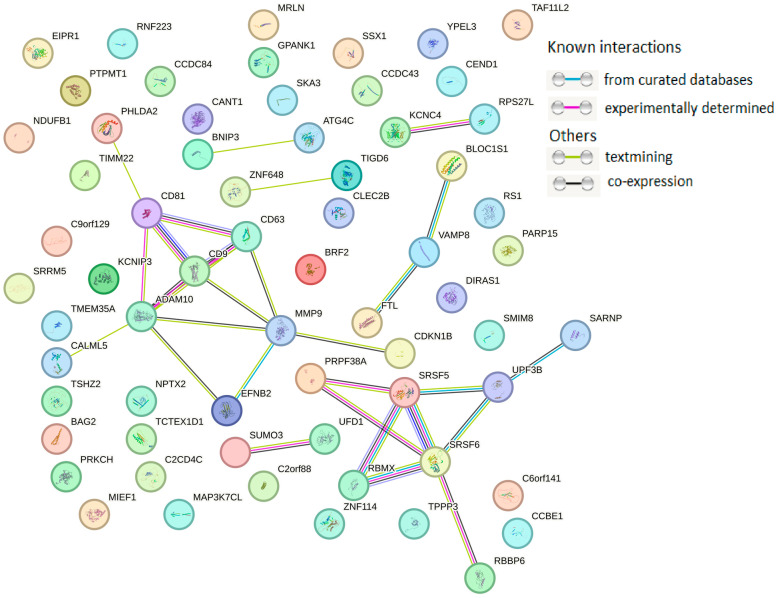
PPI network of 65 proteins from cancer cell-derived exosomes. PPI networks were plotted with STRING (Available online: http://string-db.org/, accessed on 10 January 2025) with the following settings: minimum interaction score—high confidence (0.400); active interaction sources—text mining, experiments, databases, co-expression.

For cancer-derived exosomes, the PPI network was mainly concentrated on the connections among UPF3B, SRSF6, SRSF5, RBMX, PRPF38A, KCNC4, RPS27I, VAMP8, EFNB2, CD9, CD63, CD81, ADAM10, and MMP9, among which 11 exosomal proteins formed two clusters: in addition to the cluster of major proteins identified by molecular cargo analysis of primary endotheliocytes, one cluster was identified that enhanced the malignant potential of MCF-7 and BT-474 cells (Figure 5).

The molecular function GO analysis revealed that MCF-7-derived exosomal proteins were commonly enriched in functions such as “calcium ion binding”, “metallopeptidase activity”, “RNA binding”, “ubiquitin-specific protease activity”, “GTPase activity”, “DNA binding”, etc. (Table 7).

Analysis of the protein cargo of exosomes secreted by MCF-7 cells showed the involvement of proteins in biological pathways such as “membrane trafficking”, “Clathrin-derived vesicle budding”, “trans-Golgi network vesicle budding”, “signaling by SCF-KIT”, “Notch signaling pathway”, “signaling by EGFR”, etc. (Table 8). It should be noted that five of the eight (63%) proteins in Table 6 were identified by PPI analysis as proteins interacting with a large number of other proteins (Figure 5).

The molecular functions of BT-474 cell-derived exosomal proteins were very similar to the functions of proteins secreted by MCF-7 exosomes. Specifically, bioinformatics analysis showed that the most common molecular functions of exosomal proteins were “metallopeptidase activity” and “RNA binding” (Table 9).

Similarly, bioinformatics analysis of proteins from BT-474 cell-secreted exosomes revealed their involvement in biological pathways such as “gene expression”, “mRNA splicing”, “mRNA processing”, “transcription”, etc. (Table 10). As with exosomal proteins from MCF-7 cells, in exosomes from BT-474 cells, five of the eight proteins (63%) presented in Table 10 were also identified by PPI analysis as proteins that interact with a large number of other proteins (Figure 5).

It was also shown that despite the different ensembles of proteins within exosomes from MCF-7 and BT-474 cell lines, these exosomal proteins are involved in many of the same biological pathways such as “post-elongation processing of intron-containing pre-mRNA”, “mRNA 3′-end processing”, “cleavage of growing transcript in the termination region”, “post-elongation processing of the transcript”, “RNA polymerase II transcription termination”, and “receptor–ligand binding initiates the second proteolytic cleavage of Notch receptor” (Table 8 and Table 10).

### 3.4. Comparative Proteomic Analysis of Exosomes in the Blood of BCPs with Luminal A and Triple-Positive Subtypes

**Figure 6:**
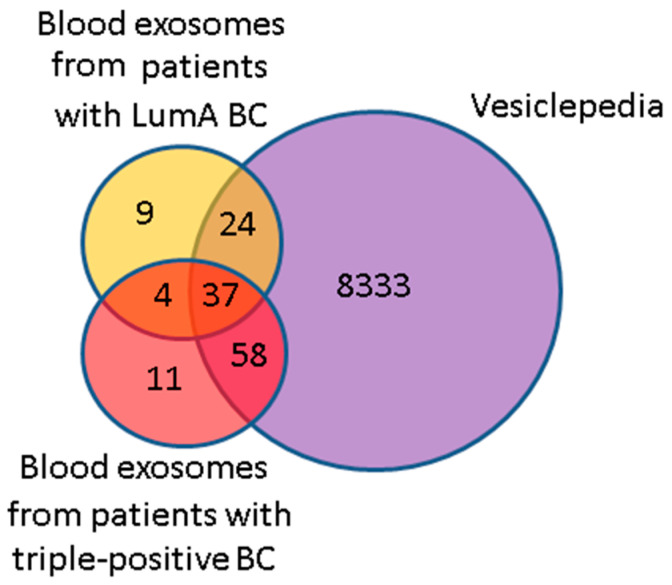
Venn–Euler diagram of proteins in exosomes from the blood of BCPs with luminal A and triple-positive subtypes and those in the Vesiclepedia database.

Using MALDI-TOF mass spectrometry with high confidence (*p* < 0.05), we identified 74 and 110 proteins in exosomes from the blood of untreated BCPs with luminal A (n = 5, Table 1) and triple-positive (n = 8, Table 1) subtypes, respectively (Supplementary Tables S4 and S5). It should be noted that 8 out of 41 universal exosomal proteins were detected in half of the samples. The analysis of the identified exosomal proteins using the Vesiclepedia database revealed that among the 119 proteins that were previously found to be associated with vesicles, a total of 61 and 95 proteins were identified in the luminal A BC and triple-positive BC exosomes, respectively. Thus, 17% of proteins identified in exosomes from the blood of BCPs in this study were not previously annotated in the Vesiclepedia database (Figure 6). The non-deposited Vesiclepedia database proteins of BCP blood exosomes are presented in Table 11.

The molecular functions of BCP blood exosome proteins were similar to those of proteins within exosomes secreted by MCF-7 and BT-474 cells. In particular, bioinformatics analysis showed that the universal molecular functions of exosomal proteins were “calcium ion binding”, “DNA binding”, “metallopeptidase activity”, and “RNA binding” (Table 7, Table 9, and Table 12).

At the same time, the bioinformatic analysis also revealed terms unique to BCP blood exosome proteins such as “protease inhibitor activity”, “protein binding”, etc.

It should be noted that barrier-to-autointegration factor (BANF1) and zinc finger protein 638 (ZNF638) were found earlier as DNA-binding proteins in exosomes [20]. In general, these exosomal DNA-binding proteins have many molecular functions and are involved in important biological processes [21,22,23,24]. Thus, exosomes carry biologically active DNA-binding proteins, mainly in the form of internal contents from donor cells to recipient cells, causing changes in the behavior of recipient cells.

**Figure 7:**
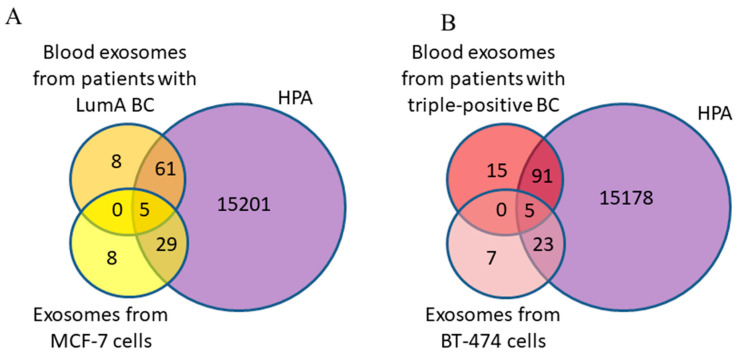
Venn–Euler diagram of exosomal proteins from cancer cells and blood samples from BCPs with luminal A (**A**) and triple-positive (**B**) subtypes compared with the HPA database, composed using QuickGO 2.0 and FunRich 3.13 software.

To determine whether the exosomal proteins we identified were found in breast neoplasms, the list of proteins was analyzed using FunRich 3.13 software to search a publicly available database for proteins in BC, namely, the Human Protein Atlas (HPA). It was shown that 66 BC-associated proteins from blood exosomes of BCPs with a luminal A subtype and 34 from MCF-7-derived exosomes were annotated in the HPA database; all 5 major exosomal proteins were also found in the HPA database (Figure 7A).

Similar results were obtained for protein cargo of exosomes from the blood of triple-positive BCPs and from BT-474 conditioned medium (Figure 7B). Thus, more BC-associated proteins were found in the composition of exosomes from the blood of BCPs than in the exosomes from the conditioned medium of cells mimicking the corresponding cancer subtype (89% and 81% for luminal A BC and MCF-7 cells and 86% and 80% for triple-positive BC and BT-474 cells, respectively).

## 4. Discussion

Tumor exosomes able to transport biologically active molecules (RNA, proteins, and metabolites) in recipient cells have been recognized as fundamental mediators of cell-to-cell communication in cancer, including BC. Since the molecular cargo of exosomes reflects the composition of the parent cell, in the field of molecular diagnostics, great potential is associated with the identification of tumor-specific signatures in the composition of exosomes for the development of a method for the diagnosis of malignant neoplasms by liquid biopsy [25,26]. One of the undoubted advantages of exosomal proteome research is the possibility of removing ballast proteins from blood plasma and increasing the concentration of tumor-specific proteins, including membrane proteins. It should be noted that the membrane of vesicles protects their contents from the action of proteases and nucleases, and vesicle preparations are stable and can be stored for a long time in laboratory conditions [27].

The search for tumor markers in exosomes is complicated by the high individual variability of the exosome proteome, even in the group of healthy donors. In a comparison of the proteomic profiles of blood plasma exosomes from fifteen clinically healthy donors, only 9 out of 109 identified exosomal proteins were present in all samples [28]. Our profiling of the proteome of HUVEC-derived exosomes partially confirms these data: only 29% of proteins within primary endotheliocytes were common to the three human umbilical vein donors.

To avoid the problem of low reproducibility between different samples, as well as to solve the problem of the amount of protein needed for mass spectrometry, most researchers work with cell culture exosomes, which is reflected in the Vesiclepedia database. As a result, the authors provide data on thousands of proteins in the composition of exosomes obtained from the conditioned media of various cell lines. In particular, Altelaar’s group successfully identified exosome proteomes from cell lines mimicking triple-negative (BT-549, Hs578T, LM2, MDA-MB-231), HER-2-positive (HCC1419, HCC1954, JIMT1, SKBR-3), and luminal A (MCF-7) BC subtypes [29], identifying 4648 proteins. However, the authors did not take into account the fact that if the size of exosomes is less than 150 nm, the vesicle cannot contain more than 100 proteins. Thus, there is no point in obtaining excessively large quantities of exosomes and describing proteins that can be detected in blood exosomes with very low probability.

Modern BC diagnostic methods (mammography, breast ultrasound, and MRI) can detect neoplasms larger than 0.5 cm; however, these tests are often ambiguous and have documented drawbacks [3]. For simplicity of calculation, we assume that only the surface cells of the tumor secrete exosomes to the external space. Then, considering that the surface area of a 5 mm diameter sphere-shaped tumor is 78.5 mm^2^, and taking into account the average diameter of a breast carcinoma cell of 20 µm, a quarter of a million cells will be located on the outer surface of the tumor. Taking into account the above calculations, in the current work, for the analysis of exosome proteins by mass spectrometry, the proteins in exosomes secreted by 500,000 MCF-7 or BT-474 cells were applied to a gel and separated. The obtained data on the proteomic profiles of exosomes from the conditioned media of breast carcinoma lines correspond to the number of exosomes secreted by the tumor at stage T1. A comparative analysis of proteins of secreted by primary endotheliocyte exosomes and breast carcinoma cell exosomes showed that it is possible to neglect the contribution of the endotheliocyte exosome proteome when searching for tumor markers in the composition of blood exosomes. Moreover, since the five proteins CD9, CD24, CD63, CD81, and MMP9 are universal and present in exosomes secreted by both primary endotheliocytes and breast carcinoma cells, they cannot be part of the diagnostic panels being developed.

To confirm that EVs are ideal diagnostic tools, Hoshino’s group analyzed the proteomes of EVs from different sources. It was shown that EV proteins from human plasma overlapped best with human serum-derived EVs (r^2^ = 0.92), followed by human bone marrow (r^2^ = 0.65) and lymphatic fluid EVs (r^2^ = 0.64); these proteins correlated least with human cell line- (r^2^ = 0.15) and tissue explant-derived EVs (r^2^ = 0.24), suggesting that the complexity of plasma and lymph EV proteomes may drive the divergence of tissue EV proteomes [30]. Our study also revealed an extremely weak correlation between exosome proteins secreted by MCF-7 cells and blood exosomes from patients with the luminal A subtype of BC, as well as between exosome proteins secreted by BT-474 cells and blood exosomes from patients with the triple-positive subtype of BC. However, when comparing protein profiles of blood exosomes from patients and tissue proteins, a significant coincidence of proteins was found. It should be noted that breast tumors are characterized by significant variability in their cellular composition, as well as histological, expression, and genotypic heterogeneity. In particular, the intratumoral morphologic heterogeneity of invasive breast carcinoma of a nonspecific type, the most common histologic form of BC (incidence rate up to 80%), has been described [31]. As a consequence, exosomes secreted by tumor cells have a more diverse composition than exosomes originating from MCF-7 or BT-474 cells. In addition, in the body, besides cancer cells, exosomes are secreted by other cells, in particular cells from the tumor microenvironment [32]. Thus, the exosomal content in the blood of BCPs at the T1 stage reflects the cancer-associated changes occurring not only in the developing primary tumor but also the tumor microenvironment. Despite previous studies searching for protein biomarkers within exosomes secreted by breast carcinoma cell lines [11,33,34], there is no consensus on exosomal tumor markers due to limited EV proteomic datasets from human samples and appropriate controls for data analysis and interpretation.

It should be noted that only three (GTSE1, VAV3, and SOCS3) proteins identified in our study were deposited simultaneously in Vesiclepedia and were shown to be strongly associated with BC according to the HPA database. Specifically, for the G2 and S phase-expressed-1 (GTSE1) protein, its expression is known to be significantly upregulated in BC tissues and cell lines, with high levels of this protein correlating with tumor prevalence and poor prognosis [35]. It has been shown that GTSE1 promotes tumor cell proliferation by activating the AKT signaling pathway and enhances metastasis through the regulation of the epithelial–mesenchymal transition [36].

Another protein, VAV3, has been identified as a critical player in the modulation of immune responses and cancer cell behavior. VAV3 has been shown to be involved in the activation of Rho/Rac signaling pathways that regulate cytoskeleton dynamics, cell migration, and invasion. VAV3 is known to be upregulated in various types of cancer, including breast cancer [37]. VAV3 has been found to be associated with poor prognosis and aggressive breast cancer subtypes [38].

SOCS3 is a critical negative regulator of cytokine signaling pathways, primarily modulating the Janus kinase/signal transducer and activator of transcription (JAK/STAT) axis. It achieves this through direct binding to phosphorylated tyrosine residues on activated receptors, thereby inhibiting STAT activation [39]. The dysregulation of SOCS3 expression is associated with aberrant JAK/STAT signaling, contributing to the development and progression of several cancers [40]. SOCS3 functions as a tumor suppressor in many contexts, where its downregulation correlates with enhanced tumor growth, angiogenesis, and immune evasion [41]. SOCS3 also exerts broader regulatory effects on other oncogenic pathways, including PI3K/AKT and MAPK signaling, by interacting with receptor tyrosine kinases and downstream effectors. These interactions modulate cell proliferation, survival, and migration [39].

Taken together, our results support the idea that tumor-associated proteins should be sought not in exosomes secreted by cell lines but in the composition of blood exosomes from cancer patients, while the contribution of endotheliocyte exosomes to the total pool of blood exosomes can be neglected.

## Figures and Tables

**Table 1:** Clinical characteristics of untreated BCPs.

Subtype	Hormonal Status	HER2/Neo Status	Age	T	N	M	Ki-67	G	Infiltrative Ductal Carcinoma
Luminal A n = 5	ER+ PR+	Negative	61	1	0	0	10–15%	2	Yes
			61	1	0	0	12–14%	2	Yes
			56	2	0	0	5–10%	2	Yes
			59	2	0	0	5–10%	2	Yes
			61	2	1	0	5%	2	Yes
Triple-positive n = 8	ER+ PR+	Positive	52	1	0	0	10%	2	Yes
			62	1	0	0	10–15%	2	Yes
			66	1	0	0	15%	2	Yes
			68	1	0	0	5–10%	2	Yes
			69	1	0	0	5–10%	2	Yes
			44	2	0	0	5%	2	Yes
			61	2	x *	0	15–17%	2	Yes
			67	2	0	0	20–25%	2	Yes

* no information available.

**Table 2:** HUVEC-secreted exosomal proteins not deposited in the Vesiclepedia database.

Uniprot ID	Protein Name	Gene Name
A0A0A0MS01	Probable non-functional T-cell receptor gamma variable 10	TRGV10
A0A1B0GUA6	Putative coiled-coil domain-containing protein 195	CCDC195
MTRNR2L13	Humanin-like 13	S4R3P1
Q8IU53	Protein CASC2, isoforms 1/2	CASC2
Q96GV9	Macrophage immunometabolism regulator	MACIR
Q9BXU9	Calcium-binding protein 8	CALN1
Q9HBF5	Suppressor of tumorigenicity 20 protein	ST20

**Table 3:** Analysis of the molecular functions in which HUVEC-derived exosomal proteins are involved.

Molecular Functions	*p*-Value	Gene Name
Calcium ion binding	0.1514	CALN1
Carboxypeptidase activity	0.0184	CPA3
Catalytic activity	0.3793	CD81
Defense/immunity protein activity	0.0525	GNLY
Glutathione transferase activity	0.0184	GSTT2
GTPase activity	0.1790	RAB3D
Inward rectifier channel	0.0123	KCNJ5
Metallopeptidase activity	0.0035	ADAM10
		MMP9
RNA binding	0.2786	RBM10
Transporter activity	0.0902	ALB
		GABARAPL2
Ubiquitin-specific protease activity	0.2857	PSMD10

**Table 4:** Analysis of the biological pathways in which HUVEC-derived exosomal proteins are involved.

Biological Pathways	*p*-Value	Gene Name
a6b1 and a6b4 integrin signaling	0.0437	CD9
Adaptive immune system	0.0341	CD81
		PSMD10
Alpha4 beta1 integrin signaling events	0.0412	CD81
Bile acid and bile salt metabolism	0.0338	ALB
HDL-mediated lipid transport	0.0189	ALB
Lipoprotein metabolism	0.0338	ALB
Metabolism of mRNA	0.0333	PSMD10
Metabolism of RNA	0.0466	PSMD10
Osteopontin-mediated events	0.0363	MMP9
Platelet degranulation	0.0351	CD63
Recycling of bile acids and salts	0.0139	ALB
Transport of organic anions	0.0127	ALB
Transport of vitamins, nucleosides, and related molecules	0.0375	ALB

**Table 5:** MCF-7 cell-secreted exosomal proteins not deposited in the Vesiclepedia database.

Uniprot ID	Protein Name	Gene Name
P46527	Cyclin-dependent kinase inhibitor 1B	CDKN1B
P59037	Putative uncharacterized protein encoded by LINC00313	LINC00313
P61236	Protein yippee-like 3	YPEL3
Q17RP2	Tigger transposable element-derived protein 6	TIGD6
Q32M92	Uncharacterized protein C15orf32	C15orf32
Q5SZD1	Uncharacterized protein C6orf141	C6orf141
Q5T035	Putative uncharacterized protein FAM120A2P	C9orf129
Q86UT8	Centrosomal AT-AC splicing factor	CENATAC
Q8IX90	Spindle and kinetochore-associated protein 3	SKA3
Q8N111	Cell cycle exit and neuronal differentiation protein 1	CEND1
Q8N7M0	Dynein light chain Tctex-type 5	DYNLT5
Q8TF44	C2 calcium-dependent domain-containing protein 4C	C2CD4C
Q96KF7	Small integral membrane protein 8	SMIM8
Q9Y584	Mitochondrial import inner membrane translocase subunit Tim22	TIMM22

**Table 6:** BT-474 cell-secreted exosomal proteins not deposited in the Vesiclepedia database.

Uniprot ID	Protein Name	Gene Name
A0A0B4J271	T-cell receptor alpha variable 12–3	TRAV12-3
A6NLC8	TATA-box binding protein associated factor 11 like protein 2	TAF11L2
O15537	Retinoschisin	RS1
P0DMT0	Myoregulin	MRLN
P57077	MAP3K7 C-terminal-like protein	MAP3K7CL
Q16384	Protein SSX1	SSX1
Q5TZK3	No name	FAM74A4
Q5TZK3	No name	FAM74A6
Q96MW1	Coiled-coil domain-containing protein 43	CCDC43
Q9BZI7	Regulator of nonsense transcripts 3B	UPF3B
Q9HAW0	Transcription factor IIIB 50 kDa subunit	BRF2

**Table 7:** Analysis of the molecular functions in which MCF-7 cell-derived exosomal proteins are involved.

Molecular Functions	*p*-Value	Gene Name
Acyltransferase activity	0.1879	BLOC1S1
Auxiliary transport protein activity	0.5822	VAMP8
Calcium ion binding	0.0122	CCBE1
		KCNIP3
		NPTX2
Catalytic activity	0.7539	CD81
Cell adhesion molecule activity	0.6068	MPZ
Defense/immunity protein activity	0.1467	ORM1
DNA binding	0.5097	ZNF114
		ZNF648
Endopeptidase activity	0.0052	ATG4C
GTPase activity	0.1129	DIRAS1
		GEM
Hydrolase activity	0.4114	CANT1
Isomerase activity	0.1104	PPIA
Kinase regulator activity	0.0580	CDKN1B
Metallopeptidase activity	0.0282	ADAM10
		MMP9
Protein serine/threonine kinase activity	0.5453	PRKCH
Protein tyrosine/serine/threonine phosphatase activity	0.0917	PTPMT1
Receptor signaling complex scaffold activity	0.5698	BAG2
Ribonuclease activity	0.1173	REXO2
RNA binding	0.2453	CSTF1
		SRSF6
Structural constituent of ribosome	0.3272	RPS27
Structural molecule activity	0.5052	TEKT3
Transcription factor activity	0.8934	CRX
Transcription regulator activity	0.8905	PARP15
Transporter activity	0.7813	TIMM22
Ubiquitin-specific protease activity	0.2559	COPS5
		RBBP6
Voltage-gated ion channel activity	0.2873	KCNC4

**Table 8:** Analysis of the biological pathways in which MCF-7 cell-derived exosomal proteins are involved *.

Biological Pathways	*p*-Value	Gene Name
AKT phosphorylates targets in the cytosol	0.0241	CDKN1B
Clathrin-derived vesicle budding	0.0035	VAMP8
		BLOC1S1
*Cleavage of growing transcript in the termination region*	*0.0058*	*SRSF6*
Integrins in angiogenesis	0.0126	MMP9
Membrane trafficking	0.0210	VAMP8
		BLOC1S1
*mRNA 3′-end processing*	*0.0037*	*SRSF6*
mRNA splicing	0.0330	*SRSF6*
mRNA splicing—major pathway	0.0330	*SRSF6*
Notch signaling pathway	0.0260	*ADAM10*
		CDKN1B
Notch-mediated HES/HEY network	0.0260	*ADAM10*
		CDKN1B
Platelet activation, signaling, and aggregation	0.0056	CD63
		PRKCH
Platelet degranulation	0.0025	CD63
*Post-elongation processing of intron-containing pre-mRNA*	*0.0037*	*SRSF6*
*Post-elongation processing of the transcript*	*0.0058*	*SRSF6*
*Receptor–ligand binding initiates the second proteolytic cleavage of Notch receptor*	*0.0241*	*ADAM10*
Response to elevated platelet cytosolic Ca^2+^	0.0048	CD63
RNA polymerase II transcription	0.0297	*SRSF6*
*RNA polymerase II transcription termination*	*0.0058*	*SRSF6*
Signaling by EGFR	0.02810.0281	*ADAM10*
		CDKN1B
Signaling by SCF-KIT	0.0133	CDKN1B
		MMP9
Trans-Golgi network vesicle budding	0.0035	VAMP8
		BLOC1S1

* biological pathways universal to exosomal proteins secreted by cancer cells are denoted in italics.

**Table 9:** Analysis of the molecular functions in which BT-474 cell-derived exosomal proteins are involved.

Molecular Functions	*p*-Value	Gene Name
Calcium ion binding	0.2343	CALML5
Catalytic activity	0.5393	CD81
Cell adhesion molecule activity	0.4032	CLEC2B
Metallopeptidase activity	0.0092	ADAM10
		MMP9
Oxidoreductase activity	0.2072	NDUFB1
Receptor binding	0.1696	EFNB2
RNA binding	0.0151	RBMX
		SRSF5
		UPF3B
Storage protein	0.0043	FTL
Transcription factor activity	0.7099	BRF2
Transcription regulator activity	0.7055	SSX1
Ubiquitin-specific protease activity	0.4213	SUMO3

**Table 10:** Analysis of the biological pathways in which BT-474 cell-derived exosomal proteins are involved *.

Biological Pathways	*p*-Value	Gene Name
*Cleavage of growing transcript in the termination region*	*0.0134*	SRSF5
		UPF3B
CXCR4-mediated signaling events	0.0424	MMP9
Formation and maturation of mRNA transcript	0.0396	SRSF5
		RBMX
		UPF3B
Gene expression	0.0037	SRSF5
		RBMX
		UPF3B
Hypoxic and oxygen homeostasis regulation of HIF-1α	0.0426	BNIP3
*mRNA 3′-end processing*	*0.0085*	SRSF5
		UPF3B
mRNA processing	0.0260	SRSF5
		RBMX
		UPF3B
mRNA splicing	0.0094	SRSF5
		RBMX
		UPF3B
*Post-elongation processing of intron-containing pre-mRNA*	*0.0085*	SRSF5
		UPF3B
*Post-elongation processing of the transcript*	*0.0134*	SRSF5
		UPF3B
Processing of capped intron-containing pre-mRNA	0.0186	SRSF5
		RBMX
		UPF3B
*Receptor–ligand binding initiates the second proteolytic cleavage of Notch receptor*	*0.0366*	*ADAM10*
Respiratory electron transport	0.0388	NDUFB1
*RNA polymerase II transcription termination*	*0.0134*	SRSF5
		UPF3B
Transcription	0.0359	BRF2
		SRSF5
		UPF3B
Transport of mature mRNA derived from an intron-containing transcript	0.0185	SRSF5
		UPF3B

* biological pathways universal to exosome proteins secreted by cancer cells are highlighted in italics.

**Table 11:** BCP blood exosomal proteins not deposited in the Vesiclepedia database.

Uniprot ID	Protein Name	Gene Name	BC Subtype
A0A1B0GVM6	Uncharacterized protein C11orf97	C11orf97	TP
A0A589	T-cell receptor beta variable	TRBV4-3	Lum A
O14543	Suppressor of cytokine signaling 3	SOCS3	TP
O43557	Tumor necrosis factor ligand superfamily member 14	TNFSF14	Lum A
O60397	Putative cytochrome c oxidase subunit 7A3, mitochondrial	COX7A2P2	TP
P0CE72	Oncomodulin-1	OCM	Lum A
P0DUQ2	PRAME family member 9	PRAMEF9	Lum A
P16233	Pancreatic triacylglycerol lipase	PNLIP	Lum A
P48167	Glycine receptor subunit beta	GLRB	Lum A
P49761	Dual specificity protein kinase CLK3	CLK3	Lum A&TP
P56373	P2X purinoceptor 3	P2RX3	Lum A&TP
Q13424	Alpha-1-syntrophin	SNTA1	Lum A
Q15776	Zinc finger protein with KRAB and SCAN domains 8	ZKSCAN8	Lum A&TP
Q2M218	Zinc finger protein 630	ZNF630	TP
Q52M93	Zinc finger protein 585B	ZNF585B	TP
Q53FZ2	Acyl-coenzyme A synthetase ACSM3, mitochondrial	ACSM3	TP
Q5CZ79	Ankyrin repeat domain-containing protein 20B	ANKRD20A8P	TP
Q5VZ18	SH2 domain-containing adapter protein E	SHE	TP
Q7RTT3	Putative protein SSX9	SSX9P	TP
Q8IUS5	Epoxide hydrolase 4	EPHX4	TP
Q8NDD1	Uncharacterized protein C1orf131	C1orf131	Lum A
Q8NEY8	Periphilin-1	PPHLN1	TP
Q9H4Q4	PR domain zinc finger protein 12	PRDM12	Lum A
Q9Y4E5	E3 SUMO-protein ligase ZNF451	ZNF451	Lum A&TP

**Table 12:** Analysis of the molecular functions in which BCP blood exosomal proteins are involved.

Molecular Functions	*p*-Value	Gene Name
Amidinotransferase activity	0.0204	GATM
ATPase activity	0.5344	KIF20B
Calcium ion binding	0.3618	KCNIP3
		OCM
Catalytic activity	0.0298	CD81
		EHHADH
		ACADM
		CKMT2
		HIF1AN
		HMOX1
		MMAB
		MTHFD1
Cell adhesion molecule activity	0.9153	MAEA
Chaperone activity	0.5802	TOR3A
Complement activity	0.0156	CLU
		C3
Complement binding	0.0204	CFH
Cysteine-type peptidase activity	0.2711	CAPN2
Cytokine activity	0.5213	IL16
Cytoskeletal protein binding	0.4409	SPTBN2
		ERC2
Defense/immunity protein activity	0.0654	AMBP
		AHSG
DNA binding	0.2907	BANF1
		ZNF638
		FAM50A
		ZNF585B
		ZNF630
		PRDM12
DNA-directed RNA polymerase activity	0.1806	POLR2D
Extracellular ligand-gated ion channel activity	0.2456	P2RX3
GTPase activator activity	0.6293	RABGAP1L
GTPase activity	0.7842	RAB24
Guanyl-nucleotide exchange factor activity	0.5376	VAV3
Heat shock protein activity	0.0146	CRYAA
		CRYAB
Hydrolase activity	0.7538	HAGH
Intracellular ligand-gated ion channel activity	0.1863	GLRB
Ligase activity	0.1786	ACSM3
		FARSB
Lipase activity	0.1223	PNLIP
Metallopeptidase activity	0.1520	ADAM10
		BMP1
Motor activity	0.1019	KIF3B
		KIFC3
Phospholipase activity	0.2660	PLB1
Protease inhibitor activity	0.0040	A2M
		SERPI
		NA1
		ITIH4
		SERPINB7
Protein binding	0.1290	FGG
		FGB
		FGA
Protein serine/threonine kinase activity	0.6131	ATR
		MYO3B
Protein serine/threonine phosphatase activity	0.2610	PPM1A
Protein threonine/tyrosine kinase activity	0.1750	CLK3
Protein tyrosine kinase activity	0.2298	ZAP70
Receptor activity	0.9182	IGF2R
Receptor binding	0.2208	BMP7
		TNFSF14
Receptor signaling complex scaffold activity	0.3787	SOCS3
		SNTA1
		FBF1
RNA binding	0.9210	SKIV2L
Structural constituent of cytoskeleton	0.2410	GSN
		PCNT
Structural constituent of ribosome	0.0864	RPL28
		MRPL52
		UBA52
Structural molecule activity	0.1132	KRT1
		KRT6A
		KRT6B
		PPHLN1
Transcription factor activity	0.9810	PIBF1
		ZKSCAN8
Transcription regulator activity	0.9281	PHB2
		ZNF451
		ZNF622
Transporter activity	0.0002	ABCB7
		ALB
		APOA1
		COG4
		EXOC8
		HBB
		HP
		HPX
		ITPR2
		RANBP3
		TTR
		TF
		VPS13A
Voltage-gated ion channel activity	0.2233	CACNA1A
		CACNG8

## Data Availability

The data presented in this study are available on request from the corresponding author.

## Supplementary Materials

The following supporting information can be downloaded at: https://www.mdpi.com/article/10.3390/diagnostics15081028/s1, Table S1: Proteins identified in exosomes secreted by HUVECs; Table S2: Proteins identified in exosomes secreted by BT-474 cells; Table S3: Proteins identified in exosomes secreted by MCF-7 cells; Table S4: Exosomal proteins identified in the blood of luminal A BCPs; Table S5: Exosomal proteins identified in the blood of triple positive BCPs.
