# Supplementary material for: From Cell Lines to Patients: Dissecting the Proteomic Landscape of Exosomes in Breast Cancer
Source: Diagnostics (Basel). 2025 Apr 17;15(8):1028. doi: 10.3390/diagnostics15081028 (PMC12026271; doi:10.3390/diagnostics15081028)
Supplement: Supplementary file 1 [file diagnostics-15-01028-s001.zip › Table_S1.pdf]

SUPPL Table S1. Proteins identified in exosomes secreted by HUVECs.

Gene	UniProt	Name	Peptides	Cover, %	Peptides
CD9	P21926	CD9 antigen	2	63	FDSQTKSIFEQETNNNNSSFYTGVYILIGAGALMMLVGFLGCCGAVQESQCMLGLFFGFLLVIFAIEIAAAIWGYSHKDEVIKEVQEFYKDTYNKLKTKDEPQRETLKAIHYALNCCGLAGGVEQFISDICPK<br><br>FHIIGAVGIGIAVVMIFGMIFSMILCCAIR
CD63	P08962	CD63 antigen	4	58	MSRGLQLLLLSCAYSLAPATPEVK<br><br>SEDVDLPCTAPWDPQVPYTVSWVKLLEGGEERMETPQEDHLRGQHYHQKGQNGSFDAPNERPYSLKIR<br><br>VILRVTGCPAQRKEETFKKYR<br><br>AGMERAFLPVTSPNKHLGLVTPHK
CD81	P60033	CD81 antigen	3	57	MGVEGCTKCIKYLLFVFNFVFWLAGGVILGVALWLRHDPQIGIAVVMIFGDK<br><br>DQIAKDVKQFYDQALQQAVVDDDAIRHETLDCCGSSTLTALTTSVLK<br><br>IDDLFSGKLYLIGIAAIVVAVIMIFEMILSMVLCCGIR
ADAM10	O14672	Disintegrin and metalloproteinase domain-containing protein 10	3	73	HQRAKRAVSHEDQFLRLDFHAHGRHFNLRMKRDTSLFSDEFKVETSNKVLDYDTSHIYTGHIYGEEGSFSHGSVIDGR<br><br>GGTFYVEPAERYIKDRTLPFHSVIYHEDDINYPHKYGPQGGCADHSVFER<br><br>KKRTTSAEKNTCQLYIQTDHLFFKYYGTREAVIAQISSHVKAIDTIYQTTDFSGIRNISFMVKRIRINTTADEKDPTNPFRFPNIGVEKFLELNSEQNHDDYCLAYVFTDRDFDDGVLGLAWVGAPSGSSGGICEK
MMP9	P14780	Matrix metalloproteinase-9]	6	51	MSLWQPLVLVLLVLGCCFAAPR<br><br>YGYTRVAEMRGESKSLGPALLLLQKQLSLPETGELDSATLKAMRTPR<br><br>DADIVIQFGVAEHGDGYPFDGK<br><br>GVVVPTRFGNADGAACHFPFIFEGRSYSACTTDGR

					FGFCPSERLYTQDGNADGK<br>ADSTVMGGNSAGELCVFPFTFLGKEYST
GSTT2	P0CG29	Glutathione S-transferase theta-2	3	24	MGLELFLDLVSQPSRAVYIFAKK<br>GQHKSKEFLQINSLGK<br>TLPTPSPEAYQAMLLRIARIP
GNLY	P22749	Granulysin	3	37	TQELGRDYRTCLTIVQK<br>SVSNAATRVCRTGR<br>RYQSRVTQGLVAGETAQQICEDLR
FAM24B	Q8N5W8	Protein FAM24B	2	28	IHNALKAAKEPEAVAVK<br>VWWAKNSQAK
	Q8IZM0	Putative CNGA1-overlapping antisense gene protein	2	35	MDSYSAKIR<br>ICFPFSSSIVLASGYSVRASMR
KCNJ5	P48544	G protein-activated inward rectifier potassium channel 4	4	18	KPRQRYMEKSGKCNVHHGNVQETYR<br>NSHIVEASIRAKLIK<br>SSYMDTEVLWGHRFTPVLTLEK<br>GLGGSREAR
CCDC195	A0A1B0GUA6	Putative coiled-coil domain-containing protein 195	2	10	MKLTASSQR<br>AVSFLLPMDMSSYSK
RAB3D	O95716	Ras-related protein Rab-3D	2	10	TYSWDNAQVILVGNK<br>VVPAEDGRR
MACIR	Q96GV9	Macrophage immunometabolism regulator	2	4	SSRLYKTRS<br>MPSSGDKTK
ALB	P02768	Albumin	11	35	DAHKSEVAHR<br>SLHTLFGDKLCTVATLR<br>LVRPEVDVMCTAFHDNEETFLKKYLYEIAR<br>ASSAKQRLK<br>AWAVAR<br>DVFLGMFLYEYARRHPDYSVVLLLR<br>VFDEFKPLVEEPQNLIK<br>FQNALLVR

					KVPQVSTPTLVEVSR<br>TPVSDR<br>RPCFSALEVDETYVPKEFNAETFTFHADICTLSEKER<br>KQTALVELVK<br>AVMDDFAAFVEKCCK<br>ETCFAEEGK
CALN1	Q9BXU9	Calcium-binding protein 8	4	23	MRLPEQPGEGKPENEK<br>SLGYMPSEVELAIIMQR<br>SLICAFAMAFIISVMLIAANQILRSGME
ST20	Q9HBF5	Suppressor of tumorigenicity 20 protein	2	40	LTATSVSQVQENGFVK<br>LEPKSGWMTFLEVTGK
CASC2	Q8IU53	Protein CASC2, isoforms 1/2	2	39	DVGTCRGRQMEIQK<br>LQTHTTPQIYTQLKGK
GABARAPL2	P60520	Gamma-aminobutyric acid receptor-associated protein-like 2	3	41	KWMFKEDHSLEHR<br>VSGSQIVDIDK<br>AIFLFVDKTVPQSSLTMGQLYEKEK
RBM10	P0DW28	Ribosome biogenesis inhibitor MINAS-60	2	24	RASMTMTTHLRSR<br>APRLSVGGGGGTGTAPPAR
IGLJ1	A0A0A0MT76	Immunoglobulin lambda joining 1	2	81	PSRLLLQPSPQRADPR<br>GFWSEPQSLCYVFGTGTK
PSMD10	O75832	26S proteasome non-ATPase regulatory subunit 10	2	16	MEGCVSNLMVCNLAYSGKLEEK<br>SLATRTDQDSR
MTRNR2L13	S4R3P1	Humanin-like 13	1	91	MDTQGFSCLLLLISEIDLSVKR
TRGV10	A0A0A0MS01	Probable non-functional T cell receptor gamma variable 10	2	14	KSIDIPCKISSTR<br>TSNKVEARK
CPA3	P15088	Mast cell carboxypeptidase A	3	9	IGSTVEDNPLYVLK<br>VYITFHSYSQMLLFPYGYTSK<br>HTS
