# Supplementary material for: From Cell Lines to Patients: Dissecting the Proteomic Landscape of Exosomes in Breast Cancer
Source: Diagnostics (Basel). 2025 Apr 17;15(8):1028. doi: 10.3390/diagnostics15081028 (PMC12026271; doi:10.3390/diagnostics15081028)
Supplement: Supplementary file 1 [file diagnostics-15-01028-s001.zip › Table_S2.pdf]

SUPPL Table S2. Proteins identified in exosomes secreted by BT-474 cells

Gene	UniProt	Name	Peptides	Cover, %	Peptides
CD9	P21926	CD9 antigen	2	60	FDSQTKSIFEQETNNNNSSFYTGVYILIGAGALMMLVGFLGCCGAVQESQCMLGLFFGFLLVIFAIEIAAAIWGYSHKDEVIKEVQEFYKDTYNKLKTKDEPQRETLKAIHYALNCCGLAGGVEQFISDICPK<br><br>FHIIGAVGIGIAVVMIFGMIFSMILCCAIR
CD63	P08962	CD63 antigen	4	56	GLQLLLLSCAYSLAPATPEVK<br><br>SEDVDLPCTAPWDPQVPYTVSWVKLLEGGEERMETPQEDHLRGQHYHQKGQNGSFDAPNERPYSLKIR<br><br>VILRVTGCPAQRKEETFKKYR<br><br>AGMERAFLPVTSPNKHLGLVTPHK
CD81	P60033	CD81 antigen	3	71	MGVEGCTKCIKYLLFVFNFVFWLAGGVILGVALWLRHDPQTTNLLYLELGDK<br><br>DQIAKDVKQFYDQALQQAVVDDDANNAKAVVKTFHETLDCCGSSTLTALTTSVLK<br><br>IDDLFSGKLYLIGIAAIVVAVIMIFEMILSMVLCCGIR
ADAM10	O14672	Disintegrin and metalloproteinase domain-containing protein 10	3	82	HQRAKRAVSHEDQFLRLDFHAHGRHFNLRMKRDTSLFSDEFKVETSNKVLDYDTSHIYTGHIYGEEGSFSHGSVIDGR<br><br>GGTFYVEPAERYIKDRTLPFHSVIYHEDDINYPHKYGPQGGCADHSVFER<br><br>KKRTTSAEKNTCQLYIQTDHLFFKYYGTREAVIAQISSHVKAIDTIYQ

					TTDFSGIRNISFMVKRIRINTTADEKDPTNPFRFPNIGVEKFLELNSEQNHDDYCLAYVFTDRDFDDGVLGLAWVGAPSGSSGGICEK
MMP9	P14780	Matrix metalloproteinase-9	6	53	MSLWQPLVLVLLVLGCCFAAPR<br><br>YGYTRVAEMRGESKSLGPALLLLQKQLSLPETGELDSATLKAMRTPR<br><br>DADIVIQFGVAEHGDGYPFDGK<br><br>GVVVPTRFGNADGAACHFPFIFEGRSYSACTTDGR<br><br>FGFCPSERLYTQDGNADGK<br><br>ADSTVMGGNSAGELCVFPFTFLGKEYST
UFD1	Q92890	Ubiquitin recognition factor in ER-associated degradation protein 1	5	14	MFSFNMFDHPIPR<br><br>SDVEK<br><br>IYELRVMETKPDK<br><br>LDGK<br><br>GVEPSPSPIKPGDIKRGIPNYEFK
SRRM5	B3KS81	Serine/arginine repetitive matrix protein 5	6	4	VSTDTR<br><br>GRTPGRRGSR<br><br>SSK<br><br>SYGRPRTSNRER<br><br>SHSWK<br><br>SSSK<br><br>TPSK
EFNB2	P52799	Ephrin-B2	7	19	DSVWK<br><br>VDSK<br><br>VYMVDKDQADRCTIK<br><br>DYYIISTSNGSLEGLDNQEGGVCQTR

					DPTR<br><br>RRHRK
GPANK1	O95872	G patch domain and ankyrin repeat-containing protein 1	6	24	ERK<br><br>HGQGRSLEAEDKMTHR<br><br>DAFWWTPLMCAAR<br><br>DAAQLAEEAGFPEVARMVRESHGETRSPENR<br><br>DRAWER
SRSF5	Q13243	Serine/arginine-rich splicing factor 5	4	15	FSSR<br><br>SRSRTRSSSRSR<br><br>SRK<br><br>SVSRSPVPEK
BNIP3	Q12983	BCL2/adenovirus E1B 19 kDa protein-interacting protein 3	4	8	SSSK<br><br>EFLFK<br><br>RTATLSMRNTSVMKK<br><br>GGIFSAEFLK
TRAV12-3	A0A0B4J271	T cell receptor alpha variable 12-3	2	38	QYSRKGPELLMYTYSSGNK<br><br>SSKYISLFIR
TAF11L2	A6NLC8	TATA-box binding protein associated factor 11 like protein 2	3	36	ETGRQTGVSAEMFAMPRDLK<br><br>KER<br><br>MTTLLSAMSEEQLSRYEVCRRSAFPKACIAGLMR
SUMO3	P55854	Small ubiquitin-related modifier 3	2	30	MSEEKPKEGVK<br><br>TENDHINLKVAGQDGSVVQFK
FAM74A4; FAM74A6	Q5TZK3		2	32	TWKELRNSETVPEKTWK<br><br>RHGESSKAVHK
TPPP3	Q9BW30	Tubulin polymerization-promoting protein family member 3 (TPPP/p20)	3	40	MAASTDMAGLEESFRKFAIHGDPK<br><br>NWAKLCKDCK<br><br>AKTGGAVDRLTDTSRYTGSHKERFDESGK

TMEM35A	Q53FP2	Novel acetylcholine receptor chaperone	3	24	MASPR<br><br>LTPRLSKDAYSEMK<br><br>SSEKKPLPGNAEEQPSLYEKAPQGKVKVS
SARNP	P82979	SAP domain-containing ribonucleoprotein	3	17	KVVKITSEIPQTER<br><br>GLSSDNKPMVNLDKLK<br><br>KER
CALML5	Q9NZT1	Calmodulin-like protein 5	3	21	MAGELTPEEEAQYK<br><br>NLSEAQLRK<br><br>MLAQE
RS1	O15537	Retinoschisin	4	18	SRK<br><br>FQDSSQWLQIDLK<br><br>VISGILTQGR<br><br>LNWIYYKDQTGNNRVFYGNSDR
	Q6ZS46	Putative uncharacterized protein FLJ45840	2	17	GIKMAFLDVQSSSTPQSLPLLLFSHR<br><br>GPGGRDWLRSQSSR<br><br>ATLFGHR
MRLN	P0DMT0	Myoregulin	2	63	MTGKNWILISTTTPK<br><br>SLEDEIVGR
FTL	P02792	Ferritin light chain	3	14	MSSQIR<br><br>EGYERLLKMQNQR<br><br>MGDHLTNLHR
RBMX	P38159	RNA-binding motif protein, X chromosome	4	9	DRETNK<br><br>DYPSSRDTR<br><br>DSYSSSRSDLYSSGR<br><br>QER
PHLDA2	Q53GA4	Pleckstrin homology-like domain family A member 2	4	46	SPDEVLR<br><br>RGVLTSDRLSLFPASPR<br><br>FHSILKVDCVERTGKYVYFTIVTTDHK<br><br>RALQDFR

					QER
NDUFB1	O75438	NADH dehydrogenase [ubiquinone] 1 beta subcomplex subunit 1	3	63	MVNLLQIVR<br><br>KSDER<br><br>SMLFKRELQPSEEVTWK
CCDC43	Q96MW1	Coiled-coil domain-containing protein 43	3	17	MAAPSEVAAIAPGEGDGGGGGFGSWLDGR<br><br>DESQRK<br><br>ERDKLAKQER
MAP3K7CL	P57077	MAP3K7 C-terminal-like protein	3	22	MISTAR<br><br>EFEALTEENRTLR<br><br>LRIQYQKRQGSS
BRF2	Q9HAW0	Transcription factor IIIB 50 kDa subunit	5	31	DLCR<br><br>HSGIRAAR<br><br>EKMLSRTMQLVELANETWLVTGR<br><br>FCKLANVDLPYPASSR<br><br>SVVK<br><br>RPASPALLLPPCMLKSPK<br><br>ICPVPPVSTVTGDENISDSEIEQYLR<br><br>AQAARQAATSVPNPP
CLEC2B	Q92478	C-type lectin domain family 2 member B	7	42	MMTKHKK<br><br>LTR<br><br>CYYFSKEEGDWNSSK<br><br>NRTGQWVDGATFTKSFGMR<br><br>KWICRK
UPF3B	Q9BZI7	Regulator of nonsense transcripts 3B	7	30	MKEEKEHRPK<br><br>VTLLTPAGATGSGGGTSGDSSKGEDKQDR<br><br>VGTIDDDPEYRKFLES[illegible]<br>TPETLLEEI[illegible]

					NKQRMR<br>EEEK<br>REK<br>SDSELK<br>RPEDESGRDYRER<br>EYER<br>YEKEKTFK<br>SRNR
SSX1	Q16384	Protein SSX1	3	15	MNGDDTFAKRPR<br>MKYSEKISYVYMKR<br>IQVEHPQMTFGR<br>ERK
PRPF38A	Q8NAV1	Pre-mRNA-splicing factor 38A	4	8	MANR<br>VCDIILPRLQK<br>RSPTLR<br>SRSPR<br>SPRRHRSRSR<br>KSR
MIEF1	L0R8F8	Mitochondrial ribosome and complex I assembly factor AltMIEF1	2	28	MAPWSREAVLSLYR<br>YTDRDFYFASIR
