# Supplementary material for: From Cell Lines to Patients: Dissecting the Proteomic Landscape of Exosomes in Breast Cancer
Source: Diagnostics (Basel). 2025 Apr 17;15(8):1028. doi: 10.3390/diagnostics15081028 (PMC12026271; doi:10.3390/diagnostics15081028)
Supplement: Supplementary file 1 [file diagnostics-15-01028-s001.zip › Table_S3.pdf]

SUPPL Table S3. Proteins identified in exosomes secreted by MCF-7 cells

Gene	Name	UniProt	Peptides	Cover, %	Peptides
CD9	CD9 antigen	P21926	2	65	FDSQTKSIFEQETNNNNSSFYTGVYILIGAGALMMLVGFLGCCGAVQESQCMLGLFFGFLLVIFAIEIAAAIWGYSHKDEVIKEVQEFYKDTYNKLKTKDEPQRETLKAIHYALNCCGLAGGVEQFISDICPK<br><br>EVFDNKFHIIGAVGIGIAVVMIFGMIFSMILCCAIR
CD63	CD63 antigen	P08962	4	59	MSRGLQLLLLSCAYSLAPATPEVK<br><br>SEDVDLPCTAPWDPQVPYTVSWVKLLEGGEERMETPQEDHLRGQHYHQKGQNGSFDAPNERPYSLKIR<br><br>VILRVTGCPAQRKEETFK<br><br>YR<br><br>AGMERAFLPVTSPNKHLGLVTPHK
CD81	CD81 antigen	P60033	3	57	MGVEGCTKCIKYLLFVFNFVFWLAGGVILGVALWLRHDPQTTNLLYLELGDK<br><br>DQIAKDVKQFYDQALQQAVVDDDANNAKAVVKTFHETLDCCGSSTLTALTTSVLK<br><br>IDDLFSGKLYLIGIAAIVVAVIMIFEMILSMVLCCGIR
ADAM10	Disintegrin and metalloproteinase domain-containing protein 10	O14672	3	73	HQRAKRAVSHEDQFLRLDFHAHGRHFNLRMKRDTSLFSDEFKVETSNKVLDYDTSHIYTGHIYGEEGSFSHGSVIDGR<br><br>GGTFYVEPAERYIKDRTLPFHSVIYHEDDINYPHKYGPQGGCADHSVFER<br><br>KKRTTSAEKNTCQLYIQTDHLFFKYYGTREAVIAQISSHVKAIDTIYQTTDFSGIRNISFMVKRIRINTTADEKDPTNPFRFPNIGVEKFLELNSEQNHDDYCLAYVFTDRDFDDGVLGLAWVGAPSGSSGGICEK
MMP9	Matrix metalloproteinase-9]	P14780	4	52	MSLWQPLVLVLLVLGCCFAAPRQR<br><br>YGYTRVAEMRGESKSLGPALLLLQKQLSLPETGELDSATLKAMRTPR<br><br>DADIVIQFGVAEHGDGYPFDGK<br><br>GVVVPTRFGNADGAACHFPFIFEGRSYSACTTDGR<br><br>FGFCPSERLYTQDGNADGK<br><br>ADSTVMGGNSAGELCVFPFTFLGK

					EYST
CEND1	Cell cycle exit and neuronal differentiation protein 1	Q8N111	2	12	MESRGKSASSP<br><br>QQPPAAPTTAPAK
DYNLT5	Dynein light chain Tctex-type 5	Q8N7M0	3	17	MMSDNAK<br><br>GSISSLSNHEFWRK<br><br>QMTKTISEVIK
RNF223	RING finger protein 223	E7ERA6	2	10	MSSGQQVWHTAVPPPRR<br><br>CWARCRDWR
TIMM22	Mitochondrial import inner membrane translocase subunit Tim22	Q9Y584	3	16	RQPR<br><br>SEEQKMIEKAMESCAFK<br><br>DMGQRGMSYAK
C15orf32	Uncharacterized protein C15orf32	Q32M92	3	16	EDLHPADPQSGEGVPPNRKNTK<br><br>THLRCQSSRVDGLMLK<br><br>HLMR
CDKN1B	Cyclin-dependent kinase inhibitor 1B	P46527	3	18	MSNVR<br><br>NLFGPVDHEELTRDLEK<br><br>KWNFDFQNHKPLEGK
C9orf129	Putative uncharacterized protein FAM120A2P	Q5T035	2	20	NLTEQNSYSNIPHEGK<br><br>DSQARGTAAHWHGGHVCSPNVF WR
SMIM8	Small integral membrane protein 8	Q96KF7	2	41	EFQSPGLRGVRTTTLFR<br><br>KDLYEAIDSEGHSYMRRKTSKWD
RPS27	Ribosomal protein eS27-like	Q71UM5	2	41	DLLHPSLEEEKK<br><br>LVQSPNSYFMDVK<br><br>LTEGCSFRRK
NPTX2	Neuronal pentraxin-2	P47972	4	9	CEGLAGGK<br><br>KVAELEDEKSLLHNETSAHR<br><br>VTELER<br><br>WPVETCEER
CANT1	Soluble calcium-activated nucleotidase 1	Q8WVQ1	4	12	MPVQLSEHPEWNESMHSLR<br><br>KGYLTLSDSGDKVAVEWDK<br><br>LYVGGLGK<br><br>YSEK
PARP15	Protein mono-ADP-ribosyltransferase PARP15	Q460N3	4	5	AFLQK<br><br>ETEEK<br><br>RQMDIKNDHK

					LLFHGTDADSVPYVNQHGFNR
VAMP8	Vesicle-associated membrane protein 8	Q9BV40	3	38	MEEASEGGGNDR<br><br>VRNLQSEVEGVKNIMTQNVER<br><br>TTSQK
LINC00313	Putative uncharacterized protein encoded by LINC00313	P59037	3	38	MTTLSK<br><br>SCFVTSSEVWKR<br><br>ECLGRTSCPRLA
BLOC1S1	Biogenesis of lysosome-related organelles complex 1 subunit 1	P78537	2	15	MAPGSRGERSSFR<br><br>EHQAKQNERK
EIPR1	EARP and GARP complex-interacting protein 1	Q53HC9	2	8	TSDSKVLTCAAVWRMPK<br><br>GWDTR<br><br>FWDTRNVTEPVK
CENATAC	Centrosomal AT-AC splicing factor	Q86UT8	3	12	GHVYSRKHQR<br><br>FLVTPQDYAR<br><br>KSMVKGLDSYEEKEDK<br><br>WQSR
ZNF648	Zinc finger protein 648	Q5T619	5	13	ASWSR<br><br>YLCAHK<br><br>CPACDR<br><br>SSNLSEHQTLHTGQRPFK<br><br>LVRHQRIHTGERPFPCTQCGQAFARSSTLKRHQQIHSGEK
TIGD6	Tigger transposable element-derived protein 6	Q17RP2	5	10	KRMR<br><br>ALNLANMLGYDNFQASVGWLNR<br><br>MKRAER<br><br>CWQK<br><br>VTQTLIDSKITDFLQTK
YPEL3	Protein yippee-like 3	P61236	4	39	SFQGSQGR<br><br>YEQAFESSQKYK<br><br>YIIELNHMIKDNGWD
C2orf88	Small membrane A-kinase anchor protein	Q9BSF0	2	24	MGCMKSK<br><br>CLPRMSPVNVKEEVK
CCBE1	Collagen and calcium-binding EGF domain-	Q6UXH8	4	10	EGYIREDDGKTCTR<br><br>QTVLQLKQK

	containing protein 1				YITGDK<br><br>DGSKGERGAPGPR
SKA3	Spindle and kinetochore-associated protein 3	Q8IX90	4	11	NSMDIMKIREYFQKYGYSPR<br><br>EEPVIVTPPTK<br><br>IPSTK<br><br>HGQNIRDVSNKEN
SRSF6	Serine/arginine-rich splicing factor 6	Q13247	4	20	RTSGR<br><br>QAGEVTYADAHKERTNEGVIEFRS YSDMK<br><br>LDGTEINGRNIR<br><br>SQSRSNSPLPVPPSKARSVSPPPK
BAG2	BAG family molecular chaperone regulator 2	O95816	5	37	VEALREAATAVEQEK<br><br>QISDGER<br><br>LMGRTLTVEVSVETIRNPQQQESLK<br><br>NIENSDKAIKLLEHSKGAGSKTLQQ NAESRFN
DIRAS1	GTP-binding protein Di-Ras1	O95057	4	42	MPEQSNDYRVVVFGAGGVGK<br><br>GHAFILVFSVTSKQSLEELGPIYK<br><br>CAFMETSAKMNYNVKELFQELLTL ETRRNMSLNIDGK<br><br>CTLM
KCNIP3	Calsenilin	Q9Y2W7	4	40	QPAKEVTKASDGSLLGDLGHTPLS K<br><br>QALMR<br><br>NECPTGLVDEDTFK<br><br>DGYITKEEMLAIMKSIYDMMGRHTY PILREDAPAEHVERFFEK<br><br>NQDGVVTIEEFLEACQK
C2CD4C	C2 calcium-dependent domain-containing protein 4C	Q8TF44	5	20	MRKTNMWFLERLRGSGENGAARG VGSEAGDK<br><br>LAAESKSLLK<br><br>ESLFHSEHGALAQVGSPGAGR<br><br>SVSLLK<br><br>HSVGR<br><br>GSSLKRDTLLGEK
MPZ	Myelin protein P0	P25189	6	31	YQPEGGR

					GQPYIDEVGTFKER<br><br>NPPDIVGKTSQVTLYVFEKVPTR<br><br>RLSAMEKGK<br><br>DASKR<br><br>QTPVLYAMLDHSR<br><br>GLGESRK
PTPMT1	Phosphatidylglycerophosphatase and protein-tyrosine phosphatase 1	Q8WUK0	5	59	MAATALLEAGLARVLFYPTLLYTLFRGK<br><br>DWYHR<br><br>SLTR<br><br>GVITMNEEYETRFLCNSSQEWKRLGVEQLRLSTVDMTGIPTLDNLQK<br><br>SATMVAAYLIQVHKWSPEEAVR<br><br>SYIHIRPGQLDVLK
ATG4C	Cysteine protease ATG4C	Q96DT6	6	15	DFISRIWLTYR<br><br>FTASFEASLSGER<br><br>TPTISLKETIGK<br><br>QSASMTSDNADDK<br><br>KMSFRK<br><br>FSSKEK<br><br>RFSTEEFVLL
ZNF114	Zinc finger protein 114	Q8NC26	9	39	EEWTLLDPAQR<br><br>NLAFIDWATPCKTK<br><br>VCLTSISSQHSTLREDWRCPKTEEPHR<br><br>NHSKPTCRLVPSQGDSIRQCILTRDSSIFKYNPVLNDSQK<br><br>AHNTHGREKMYDFTQCENTSR<br><br>DCQTGATSANAPNSGSHKSHCTGEK<br><br>CPECGR<br><br>HMKIHTGEKPYECGKCGK<br><br>ESSK
KCNC4	Voltage-gated potassium	Q03721	5	15	MISSVCVSSYRGRKSGNKPPSKTCLKEEMAK

	channel KCNC4				STLRTLPGTR<br><br>MWALFEDPYSSR<br><br>VGNITSVHFR<br><br>SEETSPR<br><br>RADSK<br><br>NKKAAACFLLSTGDYACADGSVR
C6orf141	Uncharacterized protein C6orf141	Q5SZD1	5	61	MNDPFARMETRGPQGAANPMDSSR<br><br>GAPLAPGARNPATAGASRSQGGGHEDRTADR<br><br>EKVLFLLHPERWLGTRGDPAR<br><br>DAADPPKYVLVRVEDYQVTQEVLQTSWAKGRMTTRTEEHFVTALTFRSSR<br><br>TGASRVHAAGRRVSPSPGTWLEEIK
PRKCH	Protein kinase C eta type	P24723	8	26	SSGTMK<br><br>VRIGEAVGLQPTRWSLRHSLFK<br><br>DRIFKHFTR<br><br>VHQINGHKFMATYLRQPTYCSHCREFIWGVFGK<br><br>TLAGMGLQPGNISPTSKLVSRSTLR<br><br>ESSK<br><br>KDVILQDDDVECTMTEKR<br><br>LFFVMEFVNGGDLMFHIQK<br><br>FDEAR<br><br>DLKLDNVLLDHEGHCKLADFGMCK<br><br>NPTMRLGSLTQGGEHAILR
TSHZ2	Teashirt homolog 2	Q9NRE2	18	17	EEEEIK<br><br>SVCGRDASDK<br><br>LSNSER<br><br>DKLRPTSYSKPR<br><br>AFQDMDKEDAQK<br><br>TKHYQK<br><br>MVTPAKK

					VTSSASKK<br><br>EEDLEDGSKGGGDILKSLENTVTTAINK<br><br>VMPLVSMPTHLAPYTQVK<br><br>ESEDK<br><br>EEEK<br><br>SASVSRR<br><br>VLPKATTPKPASSSRVPPMKLEMDVR<br><br>FTGLSMTTISHWLANVK<br><br>VEQEISR<br><br>LCCR
RBBP6	E3 ubiquitin-protein ligase RBBP6	Q7Z6E9	23	31	FSSKLNYDTVTFDGLHISLCDLK<br><br>STSKTYVISRTEPAMATTK<br><br>NFESGPR<br><br>GAMLTNTGKYAIPTIDAEAYAIGKK<br><br>DIMTDAVVIPCCGNSYCDECIRTALLESDEHTCPTCHQNDVSPDALIANKFLRQAVNNFKNETGYTK<br><br>DSDNKILPAAALASEHSKGTSSIAITALMEEKGYQVPVLGTPSLLGQSLLHGQLIPTTGPVR<br><br>RGERSCYR<br><br>SPYSGSSYSRSSYTYSKSR<br><br>SFSR<br><br>EVPPPYDMKAYYGRSVDFRDPFEK<br><br>REDYVGGQSHRSRNIGSNYPEKLSAR<br><br>SKEKESENAPGDGK<br><br>EPTGVEENKTDSLFVLPSRDDATPVRDEPMDAESITFK<br><br>ENIVKPAKGPQEKVDGERERSPR<br><br>SSSSSQKDEKITGTPR<br><br>AHSKSAKEHQETKPVKEEK

					RKTEEK<br><br>DFESSSMKISK<br><br>MEPDTEKMDRTPEK<br><br>IGSTENISNTKEPSEKLESTSSK<br><br>KVTGTEGSSSTLVDYTSTSSTGGS PVRK<br><br>ESEPSEK<br><br>SLSQSSKEARTSDK<br><br>TDYDTREYSSSKR<br><br>SNSSPSR<br><br>ATYDTK<br><br>SHSPSGSQTRSHSSSASSAESQDS KKK<br><br>EKEKDDQK
