# Supplementary material for: From Cell Lines to Patients: Dissecting the Proteomic Landscape of Exosomes in Breast Cancer
Source: Diagnostics (Basel). 2025 Apr 17;15(8):1028. doi: 10.3390/diagnostics15081028 (PMC12026271; doi:10.3390/diagnostics15081028)
Supplement: Supplementary file 1 [file diagnostics-15-01028-s001.zip › Table_S4.pdf]

SUPPL Table S4 Exosomal proteins identified in the blood of luminal A BCPs*

Gene Name	Name	UniprotID	Score
ADAM10	*Disintegrin and metalloproteinase domain-containing protein 10*	*O14672*	*60*
AHSG	*Alpha-2-HS-glycoprotein*	*P02765*	*60*
ALB	*Serum albumin*	*P02768*	*149*
AMBP	*Alpha-1-microglycoprotein*	*P02760*	*60*
APOA1	*Apolipoprotein A-I*	*P02647*	*176*
APPBP2	*Amyloid protein-binding protein 2*	*Q92624*	*61*
BANF1	*Barrier-to-autointegration factor*	*O75531*	*57*
BMP1	*Bone morphogenetic protein 1*	*P13497*	*60*
C1orf131	Uncharacterized protein C1orf131	Q8NDD1	56
C3	*Complement C3*	*P01024*	*137*
CABP1	*Calcium-binding protein 1*	*Q9NZU7*	*56*
CACNG8	*Voltage-dependent calcium channel gamma-8 subunit*	*Q8WXS5*	*63*
CCDC146	Coiled-coil domain-containing protein 146	Q8IYE0	57
CCDC152	Coiled-coil domain-containing protein 152	Q4G0S7	63
CD24	*Signal transducer CD24*	*P25063*	*60*
CD63	*CD63 antigen*	*P08962*	*60*
CD81	*CD81 antigen*	*P60033*	*60*
CD9	*CD9 antigen*	*P21926*	*60*
CKAP2L	*Cytoskeleton-associated protein 2-like*	*Q8IYA6*	*65*
CLK3	*Dual specificity protein kinase CLK3*	*P49761*	*56*
CLU	*Clusterin*	*P10909*	*60*
COG4	*Conserved oligomeric Golgi complex subunit 4*	*Q9H9E3*	*56*
COX7A2P2	Putative cytochrome c oxidase subunit 7A3, mitochondrial	O60397	58
ERC2	ERC protein 2	O15083	56
EXD3	Exonuclease mut-7 homolog	Q8N9H8	60
EXOSC7	*Exosome complex component RRP42*	*Q15024*	*56*
FBF1	Fas-binding factor 1	Q8TES7	56
FGA	*Fibrinogen alpha chain*	*P02671*	*57*
FGB	*Fibrinogen beta chain*	*P02675*	*60*
FGG	*Fibrinogen gamma chain*	*P02679*	*67*
GLRB	Glycine receptor subunit beta	P48167	56
GTSE1	G2 and S phase-expressed protein 1	Q9NYZ3	60
HAGH	Hydroxyacylglutathione hydrolase, mitochondrial	Q16775	56
HBB	*Hemoglobin subunit beta*	*P68871*	*72*
HMOX1	*Heme oxygenase 1*	*P09601*	*56*
IGHV3-74	*Immunoglobulin heavy variable 3-74*	*A0A0B4J1X5*	*56*
IGHA1	Immunoglobulin heavy constant alpha 1	P01876	58
IGHG1	Ig gamma-1 chain C region	P01857	59
IGKC	*Ig kappa chain C region*	*P01834*	*59*
IGKV3-20	Immunoglobulin kappa variable 3-20	P01619	57
IGLC7	Immunoglobulin lambda constant 7	A0M8Q6	56

KCNIP3	Calsenilin	Q9Y2W7	58
KIF20B	Kinesin-like protein KIF20B	Q96Q89	60
KRT1	Keratin, type II cytoskeletal 1	P04264	81
KRT6A	*Keratin, type II cytoskeletal 6A*	*P02538*	*62*
KRT6B	*Keratin, type II cytoskeletal 6B*	*P04259*	*58*
MIEF1	Mitochondrial dynamics protein MID51	Q9NQG6	60
MMAB	Corrinoid adenosyltransferase MMAB	Q96EY8	56
MTHFD1	C-1-tetrahydrofolate synthase, cytoplasmic	P11586	59
OCM	Oncomodulin-1	P0CE72	61
P2RX3	*P2X purinoceptor 3*	*P56373*	*56*
PCNT	Pericentrin	O95613	71
PDS5A	Sister chromatid cohesion protein PDS5 homolog A	Q29RF7	57
PNLIP	Pancreatic triacylglycerol lipase	P16233	56
POLR2D	DNA-directed RNA polymerase II subunit RPB4	O15514	56
PRAMEF9	PRAME family member 9	P0DUQ2	58
PRDM12	PR domain zinc finger protein 12	Q9H4Q4	56
RUBCN	Run domain Beclin-1-interacting and cysteine-rich domain-containing protein	Q92622	56
SERPINA1	Alpha-1-antitrypsin	P01009	56
SERPINB7	*Serpin B7*	*O75635*	*56*
SNTA1	Alpha-1-syntrophin	Q13424	60
TF	*Serotransferrin*	*P02787*	*137*
TMEM9B	Transmembrane protein 9B	Q9NQ34	63
TNFSF14	Tumor necrosis factor ligand superfamily member 14	O43557	56
TPD52L2	Tumor protein D54	O43399	61
TRANK1	*TPR and ankyrin repeat-containing protein 1*	*O15050*	*57*
TRBV4-3	T cell receptor beta variable	A0A589	56
TTR	Transthyretin	P02766	58
UBA52	Ubiquitin-60S ribosomal protein L40	P62987	57
VAV3	*Guanine nucleotide exchange factor VAV3*	*Q9UKW4*	*80*
VPS13A	*Intermembrane lipid transfer protein VPS13A*	*Q96RL7*	*58*
ZKSCAN8 ZNF192	*Zinc finger protein with KRAB and SCAN domains 8*	*Q15776*	*56*
ZNF451	*E3 SUMO-protein ligase ZNF451*	*Q9Y4E5*	*69*
ZNF622	*Cytoplasmic 60S subunit biogenesis factor ZNF622*	*Q969S3*	*57*

*Proteins common to the luminal A and triple positive subtypes, in italics
