# Supplementary material for: From Cell Lines to Patients: Dissecting the Proteomic Landscape of Exosomes in Breast Cancer
Source: Diagnostics (Basel). 2025 Apr 17;15(8):1028. doi: 10.3390/diagnostics15081028 (PMC12026271; doi:10.3390/diagnostics15081028)
Supplement: Supplementary file 1 [file diagnostics-15-01028-s001.zip › Table_S5.pdf]

SUPPL Table S5 Exosomal proteins identified in the blood of triple positive BCPs*

Gene Name	Name	UniprotID	Score
A1BG	Alpha-1B-glycoprotein	P04217	128
A2M	AP-1 complex subunit mu-2	Q9Y6Q5	59
ABCB7	Iron-sulfur clusters transporter ABCB7, mitochondrial	O75027	56
ACADM	Medium-chain specific acyl-CoA dehydrogenase, mitochondrial	P11310	58
ACSM3	Acyl-coenzyme A synthetase ACSM3, mitochondrial	Q53FZ2	56
ADAM10	*Disintegrin and metalloproteinase domain-containing protein 10*	*O14672*	*60*
AHSG	*Alpha-2-HS-glycoprotein*	*P02765*	*60*
ALB	*Serum albumin*	*P02768*	*149*
AMBP	*Alpha-1-microglycoprotein*	*P02760*	*60*
ANKRD20A8P	Ankyrin repeat domain-containing protein 20B	Q5CZ79	59
ANO3	Anoctamin-3	Q9BYT9	56
APOA1	*Apolipoprotein A-I*	*P02647*	*176*
APOA4	Apolipoprotein A-IV	P06727	60
APPBP2	*Amyloid protein-binding protein 2*	*Q92624*	*61*
APPBP2	Amyloid protein-binding protein 2	Q92624	61
ATR	Serine/threonine-protein kinase ATR	Q13535	58
BANF1	*Barrier-to-autointegration factor*	*O75531*	*57*
BMP1	*Bone morphogenetic protein 1*	*P13497*	*60*
C11orf97	Uncharacterized protein C11orf97	A0A1B0GVM6	56
C3	*Complement C3*	*P01024*	*137*
CABP1	*Calcium-binding protein 1*	*Q9NZU7*	*56*
CACNG8	*Voltage-dependent calcium channel gamma-8 subunit*	*Q8WXS5*	*63*
CCDC158	Coiled-coil domain-containing protein 158	Q5M9N0	58
CCDC85A	Coiled-coil domain-containing protein 85A	Q96PX6	64
CD24	*Signal transducer CD24*	*P25063*	*60*
CD63	*CD63 antigen*	*P08962*	*60*
CD81	*CD81 antigen*	*P60033*	*60*
CD9	*CD9 antigen*	*P21926*	*60*
CFH	Complement factor H	P08603	63
CKAP2L	*Cytoskeleton-associated protein 2-like*	*Q8IYA6*	*65*
CKMT2	Creatine kinase S-type, mitochondrial	P17540	57
CLK3	*Dual specificity protein kinase CLK3*	*P49761*	*56*
CLU	*Clusterin*	*P10909*	*60*
COG4	*Conserved oligomeric Golgi complex subunit 4*	*Q9H9E3*	*56*
COX7A2P2	Putative cytochrome c oxidase subunit 7A3, mitochondrial	O60397	58
CRYAA	Alpha-crystallin A chain	P02489	57
CRYAB	Alpha-crystallin B chain	P02511	59
DTNA	Dystrobrevin alpha	Q9Y4J8	57
EHHADH	Peroxisomal bifunctional enzyme	Q08426	56
EPHX4	Epoxide hydrolase 4	Q8IUS5	57
EXOSC7	*Exosome complex component RRP42*	*Q15024*	*56*
FAM50A	Protein FAM50A	Q14320	61
FARSB	Phenylalanine-tRNA ligase beta subunit	Q9NSD9	56
FGA	*Fibrinogen alpha chain*	*P02671*	*57*
FGB	*Fibrinogen beta chain*	*P02675*	*60*

FGG	*Fibrinogen gamma chain*	*P02679*	*67*
GATM	Glycine amidinotransferase, mitochondrial	P50440	56
GSN	Gelsolin	P06396	60
HBB	*Hemoglobin subunit beta*	*P68871*	*72*
HIF1AN	Hypoxia-inducible factor 1-alpha inhibitor	Q9NWT6	61
HMOX1	*Heme oxygenase 1*	*P09601*	*56*
HP	Haptoglobin	P00738	74
HPR	Haptoglobin-related protein	P00739	60
HPX	Hemopexin	P02790	60
IGHA1	Immunoglobulin heavy constant alpha 1	P01876	58
IGHG2	Ig gamma-2 chain C region	P01859	59
IGHV3-74	*Immunoglobulin heavy variable 3-74*	*A0A0B4J1X5*	*56*
IGKC	*Ig kappa chain C region*	*P01834*	*59*
IL16	Pro-interleukin-16	Q14005	56
ITIH4	Inter-alpha-trypsin inhibitor heavy chain H4	Q14624	80
ITPR2	Inositol 1,4,5-trisphosphate receptor type 2	Q14571	67
KRT1	Keratin, type II cytoskeletal 1	P04264	81
KDM6B	Lysine-specific demethylase 6B	O15054	57
KIF3B	Kinesin-like protein KIF3B	O15066	56
KIFC3	Kinesin-like protein KIFC3	Q9BVG8	59
KRT6A	*Keratin, type II cytoskeletal 6A*	*P02538*	*62*
KRT6B	*Keratin, type II cytoskeletal 6B*	*P04259*	*58*
LPCAT2	Lysophosphatidylcholine acyltransferase 2	Q7L5N7	56
LRG	Leucine-rich alpha-2-glycoprotein	P02750	60
MAEA	E3 ubiquitin-protein transferase MAEA	Q7L5Y9	56
MRPL52	Large ribosomal subunit protein mL52	Q86TS9	58
MYO3B	Myosin-IIIb	Q8WXR4	56
P2RX3	*P2X purinoceptor 3*	*P56373*	*56*
PCNT	Pericentrin	O95613	71
PDS5A	Sister chromatid cohesion protein PDS5 homolog A	Q29RF7	57
PDZD2	PDZ domain-containing protein 2	O15018	57
PHB2	Prohibitin-2	Q99623	74
PIBF1	Progesterone-induced-blocking factor 1	Q8WXW3	59
PLB1	Phospholipase B1, membrane-associated	Q6P1J6	56
PPHLN1	Periphilin-1	Q8NEY8	56
PPM1A	Protein phosphatase 1A	Q13522	56
RAB24	Ras-related protein Rab-24	Q969Q5	59
RABGAP1L	Rab GTPase-activating protein 1-like	Q5R372	56
RANBP3	Ran-binding protein 3	Q9H6Z4	56
RPL28	Large ribosomal subunit protein eL28	P46779	59
SACS	Sacsin	Q9NZJ4	56
SERPINB7	*Serpin B7*	*O75635*	*56*
SHE	SH2 domain-containing adapter protein E	Q5VZ18	58
SKIV2L	Helicase SKI2W	Q15477	65
SOCS3	Suppressor of cytokine signaling 3	O14543	63
SPTBN2	Spectrin beta chain, non-erythrocytic 2	O15020	66
SSX9P	Putative protein SSX9	Q7RTT3	56
TCHP	Trichoplein keratin filament-binding protein	Q9BT92	58
TF	*Serotransferrin*	*P02787*	*137*
TNFSF14	Tumor necrosis factor ligand superfamily member 14	O43557	56

TOR3A	Torsin-3A	Q9H497	63
TPD52L1	Tumor protein D53	Q16890	56
TPD52L2	Tumor protein D54	O43399	61
TRANK1	*TPR and ankyrin repeat-containing protein 1*	*O15050*	*57*
TTR	Transthyretin	P02766	58
VAV3	*Guanine nucleotide exchange factor VAV3*	*Q9UKW4*	*80*
VPS13A	*Intermembrane lipid transfer protein VPS13A*	*Q96RL7*	*58*
ZAP70	Tyrosine-protein kinase ZAP-70	P43403	59
ZKSCAN8 ZNF192	*Zinc finger protein with KRAB and SCAN domains 8*	*Q15776*	*56*
ZNF451	*E3 SUMO-protein ligase ZNF451*	*Q9Y4E5*	*69*
ZNF585B	Zinc finger protein 585B	Q52M93	57
ZNF622	*Cytoplasmic 60S subunit biogenesis factor ZNF622*	*Q969S3*	*57*
ZNF630	Zinc finger protein 630	Q2M218	80
ZNF638	Zinc finger protein 638	Q14966	59

*Proteins common to the luminal A and triple positive subtypes, in italics
